# The Thoracic Lymphatic Vasculature: Interorgan Strategies to Achieve an Optimal Tissue Function

**DOI:** 10.1002/cph4.70143

**Published:** 2026-04-12

**Authors:** Daniela Negrini, Eleonora Solari, Cristiana Marcozzi, Andrea Moriondo

**Affiliations:** ^1^ Department of Medicine and Technological Innovation (DIMIT) University of Insubria Varese Italy

**Keywords:** lacunae, loops, lymphatic flow modulation, lymphatic vasculature, stomata, unidirectional valves

## Abstract

In thoracic tissues, the lymphatic vasculature not only contributes to fluid and solute homeostasis but also plays a critical role in shaping overall tissue physiology. Although the general morphology of lymphatic vessels and their organization within the vascular circuit are largely conserved inter‐organs, thoracic lymphatics exhibit highly specialized structural features, such as the presence of stomata and large lacunae, which are exclusively found in pleural and peritoneal mesothelia. These distinct anatomical specializations characterize thoracic lymphatics, which extensively supply organs such as the lung and the heart, as well as serosal compartments including the pleural space and the suprahepatic subdiaphragmatic peritoneal regions, and are associated with equally specialized mechanisms that sustain lymph formation and propulsion. While lymph flow in all tissues may rely on spontaneous contraction of the lymphatic muscles located within the vessel wall and/or on extrinsic tissue motion, the thoracic lymphatic vasculature displays an exceptional diversity of morphological and functional solutions that allow these mechanisms to be exploited with high efficiency, in close adaptation to local anatomical and mechanical environments. Accordingly, this Review focuses on the inter‐organ strategies developed by thoracic lymphatics to match local drainage requirements, highlighting how structural specialization and mechanical integration with surrounding tissues optimize lymphatic function and, ultimately, overall tissue performance.

## Graphical abstract

In the thorax, organ‐specific features such as stomata and submesothelial lacunae, pulmonary perivascular and peribronchial vessels, and subepicardial lymphatic network allow the thoracic lymphatics to perform as a dynamically regulated system able to exploit local mechanical forces and tissue‐specific architecture to maintain fluid and solute homeostasis.

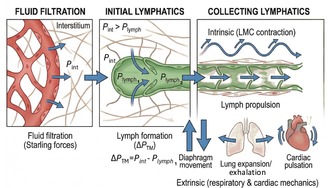

## 1 Introduction

In mammals, blood flowing in the pulmonary and systemic vasculature is confined within the surrounding extracellular environment, so that body fluids are distributed among three distinct compartments arranged in series with each other: (a) the intracellular compartment, (b) the extracellular intravascular compartment, and, between them, (c) the extracellular interstitial space. Within the latter, a flexible three‐dimensional scaffold of insoluble macromolecules, such as fibrillar collagens, elastin, hyaluronan, and proteoglycans, is bathed by interstitial fluid, which consists of soluble molecules spanning a wide range of molecular weights, from salts to plasma‐derived proteins, dispersed in the aqueous phase permeating the matrix fibers and cells. In such an environment, water is present both as a chemically bound molecule and as freely mobile fluid, whose volume varies markedly among tissues depending on their physiological or pathophysiological states. The chemically bound fraction dynamically equilibrates with free water, which continuously flows from the intravascular compartment into the surrounding interstitial space.

The three‐dimensional interstitial matrix scaffold surrounds and supports parenchymal and stromal cells and confers both soft and hard tissues with their characteristic mechanical strength and resistance to compression and expansion. In addition, in microvascular and lymphatic endothelia the matrix fibers contribute to endothelial sieving properties with respect to water and solutes. Within the cellular basement membrane and even within intercellular clefts, specific matrix macromolecules, such as collagen Type IV, perlecan, or laminins, form a dense mesh with pores of approximately 400 nm in diameter, organized in a supramolecular architecture characterized by size‐ and charge‐selective properties. Within the pool of insoluble matrix fibers, collagen VII plays a specialized role in lymphatic vascular function. Specifically, collagen VII is the major structural constituent of the so‐called “anchoring filaments,” spring‐like fibers that bridge the lamina densa of endothelial and epithelial cells to collagen Types I and IV, fibronectin, and laminin (Rousselle et al. 1997), thereby providing a mechanical linkage between the abluminal endothelial surface and the extracellular matrix scaffold. Extracellular extravascular compartments also include the internal body cavities, the so‐called “coelomic cavities,” consisting of the pleural, pericardial, peritoneal, cranial, and vertebral cavities. They are devoid of solid fibers but contain extracellular fluid, whose turnover mechanisms are analogous to those operating in all other extracellular tissue spaces. Accordingly, these cavities may be considered as “matrix‐free, fluid‐filled” extravascular tissues.

Fluid enters the perivascular interstitial tissue along combined hydraulic and colloidosmotic pressure gradients that develop between the microvascular compartment and the surrounding interstitial space (Guyton et al. 1971). The continuous entry of fluid into the interstitial space and the serosal cavities would inevitably and progressively deplete plasma volume and further increase interstitial/serosal swelling if fluid entry were not counterbalanced by its simultaneous removal. In the great majority of body tissues, such drainage occurs through the lymphatic vasculature, a closed unidirectional vascular network that belongs to the cardiovascular system and performs several essential body functions:

- it removes fluid from interstitial tissues, maintaining normal tissue hydration;
- it controls plasma volume by returning interstitial fluid to the venous circulation, thus indirectly contributing to the regulation of arterial pressure and cardiovascular function;
- it returns proteins and other molecules from the interstitial space to the blood, thus maintaining physiological tissue protein and solute concentration;
- it returns leukocytes, cell debris, and tumor cells to the bloodstream;
- it provides the main route for the transport of chylomicrons (digested and absorbed lipids) from the intestine to the blood circulation;
- it may serve as a reservoir for extracellular fluid

In addition, the dissemination of lymph nodes along the lymphatic network provides an important contribution to host immune defense by presenting antigens and antigen‐presenting cells to B and T lymphocytes within the lymph node hilum.

No lymphatics have been identified in avascular tissues, such as the epidermis, the cartilage, the cornea, and the inner layer of large arteries. Some vascularized tissues, such as bone marrow, retina, umbilical cord, and renal medulla, also essentially lack lymphatics (Aukland and Reed 1993). In these tissues, which are all characterized by elevated local interstitial fluid pressure (*P*_int_), fluid drainage takes place exclusively through the venous side of the vascular capillaries (Michel and Phillips 1987).

A special mention is warranted regarding the lymphatic function in the brain, whose parenchyma is devoid of a local “classical” lymphatic circuit. Lymphatics are nevertheless involved in the turnover of the cerebrospinal fluid (CSF) and of the brain extracellular interstitial fluid, being part of a recently described (Iliff et al. 2012) cerebral pathway, the glial‐associated lymphatic, or glymphatic, system, which supports the drainage of fluid and solutes, such as proteins or even β‐amyloids (Iliff et al. 2012) from the brain parenchyma. The pathway encompasses filtration, percolation and final drainage of the CSF: (a) at the level of the choroidal plexus epithelium, osmotic pressure gradients sustain the production of CSF which continuously enters the brain ventricles lumen through Aquaporin 1 water channels (Jessen et al. 2015); (b) hydraulic pressure gradients maintain the subsequent percolation of CSF along the arterial and arteriolar perivascular Virchow‐Robin spaces toward the pericapillary interstitium; (c) the entire arterial‐to‐capillary cerebral vasculature is bordered by astrocytes whose pedicles express Aquaporin 4 water channels (Mathiisen et al. 2010), through which a net transit of CSF from the Virchow–Robin space to the perineural brain interstitium takes place. In this way, CSF continuously blends with cerebral interstitial fluid to be then driven toward the perivascular space surrounding large cerebral veins, such as the caudal rhinal and the internal cerebral veins. Interestingly, similarly to what is observed in thoracic lymphatics, CSF production and percolation along the perivascular space are significantly enhanced by arterial and respiratory swings (Jessen et al. 2015). Final removal of CSF from the perivenous space is thought to occur through the arachnoid granulations (Pollay 2010) and/or directly into meningeal lymphatics (Aspelund et al. 2015; Zhang et al. 2025). In fact, recent studies have demonstrated the existence of an extensive network of meningeal lymphatics lining the dural sinuses (Louveau et al. 2015) and eventually discharging into the deep cervical lymph vessels. These vessels present morphological details typical of initial lymphatics, such as discontinuous endothelium and basal lamina, external anchoring filaments, lack of lymphatic muscle cells in the wall and positive staining with the specific lymphatic endothelial cell marker LYVE‐1 (Louveau et al. 2015). Through the cribriform plate and the olfactory bulb's subarachnoid space, CSF may also enter the lymphatic vessels of the nasal mucosa, which also ultimately reach the deep cervical lymph nodes (Koh et al. 2005; Zakharov et al. 2003). Such evidence not only points to the direct physiological involvement of the lymphatic system in maintaining cerebral fluid homeostasis, but also opens new perspectives on the role played by lymphatics in the development and progression of several cerebral pathologies.

Although the general structure and vessel hierarchy are similar in all tissues supplied by lymphatics, in contrast to the relatively uniform organization of the systemic circulation, significant differences in the local architecture, arrangement, and functional relevance of the lymphatic vasculature are encountered across different tissues. A remarkable example is provided by the widely variable organization of lymphatics in thoracic organs, and in the adjacent pleural and peritoneal cavities, where lymphatic drainage fulfills, in addition to its general functions, highly specific and critical functions. In particular, thoracic lymphatics play a key interorgan role in pleural fluid turnover. Intercostal lymphatics, in addition to draining interstitial fluid around skeletal muscle fibers, ensure drainage of pleural fluid facing the costal surface of the lung. Similarly, lymphatics of the convex diaphragmatic dome drain the interstitial space among skeletal muscle fibers, as well as fluid in the diaphragmatic pleural region at the apposition zone. The combined action of intercostal and pleural diaphragmatic lymphatics sustains the vast majority of the overall pleural fluid reabsorption (Miserocchi et al. 1988; Negrini et al. 1985), thereby establishing and maintaining a minimal pleural fluid volume and the subatmospheric intrapleural fluid pressure. This, in turn, directly affects lung volume during the entire respiratory cycle, with significant impact on multiple aspects of respiratory function:

- the maintenance of a normal pleural fluid volume ensures a tight lung–chest wall coupling and a subatmospheric pleural pressure (*P*_pl_) that guarantees, under normal breathing with near‐atmospheric alveolar pressure (*P*_alv_), a positive transpulmonary pressure (*P*_TP_ = *P*_alv_ − *P*_pl_), which keeps the lung expanded at end‐expiration and allows tidal lung inflation during inspiration;
- the uneven distribution of pressures within the pleural space contributes to regional differences in lung expansion, representing one of the factors responsible for the uneven ventilation/perfusion ratio;
- the presence of a thin layer of fluid in the pleural cavity ensures a tight lung–chest wall coupling, allowing lung volume to faithfully follow changes in chest wall volume throughout the entire respiratory cycle. Pleural effusion, such as that observed in cases of pleural lymphatic failure, increases intrapleural fluid volume and pressure, thereby leading to lung collapse and respiratory distress;
- by avoiding direct contact between the visceral and parietal mesothelial surfaces, pleural fluid allows an almost frictionless sliding between them, thus preventing mechanical damage;
- by affecting intrapleural pressure and lung expansion, pleural lymphatics also indirectly impact local pulmonary interstitial pressure and microvascular fluid flux, thereby modulating pulmonary lymphatic function (Miserocchi et al. 1990, 1991).

In this context, the existence of a subatmospheric intrapleural pressure influences not only lung volume, but also the volume of other distensible intrathoracic structures, such as the atria, whose inspiratory distension enhances venous return and overall cardiovascular function, and the esophagus. In addition, diaphragmatic lymphatics, along with those supplying the abdominal muscular wall, play an important role in peritoneal fluid turnover, providing, on the one hand, peritoneal fluid outflow and, on the other, a safety peritoneal‐to‐pleural drainage route in the case of significant abdominal effusion.

Given the multiple functional implications and the interorgan relevance of thoracic lymphatics, this Review focuses on their unique features, mechanisms, and physiological and pathophysiological aspects, highlighting the interaction among lymphatic networks of different thoracic organs to achieve optimal tissue and organ performance.

## 2 Fluid Balance and Lymphatic Supply in Thoracic Tissues and in Pleural‐Peritoneal Cavities

Fluid enters the interstitial space via a purely passive paracellular transport along the inter‐endothelial clefts, as originally described and quantified by Sir Ernest Starling (Starling 1896):

(1) $$J_{v}=L_{p}\cdot S\cdot \left[\left(P_{A}-P_{B}\right)-\sigma \cdot \left(\pi_{A}-\pi_{B}\right)\right]$$

where *J*_v_ is the fluid bulk flow, *L*_p_, *S* and *σ* are the hydraulic conductivity, the surface area, and the reflection coefficient for plasma proteins of the membrane through which fluid flux occurs, respectively, and *P* and π are the hydraulic and the colloidosmotic pressures in the two compartments A and B separated by the membrane (Granger et al. 1984). In the case of interstitial fluid filtration, compartments A and B are the capillary lumen and the surrounding interstitial space, separated by the endothelial wall, respectively. To produce pleural or peritoneal fluid, A and B refer to the submesothelial interstitium and the serosal cavity, separated by the pleural or peritoneal mesothelium, respectively.

While systemic/pulmonary intravascular pressures and colloidosmotic pressures are relatively constant under normal steady‐state conditions, *P*_int_ may undergo rapid and significant variations, affecting both fluid filtration and local lymph flow.

Since the original formulation of the concept of interstitial fluid pressure introduced by Arthur Guyton in the early 1960s (Guyton 1963), it has become clear that *P*_int_ is subatmospheric (approximately −6 to −8 mmHg) in most tissues (Guyton et al. 1971; Aukland and Reed 1993), particularly in thoracic ones (Miserocchi et al. 1990, 1991; Moriondo et al. 2005). For example, in the lung parenchyma at heart level, *P*_int_ varies during tidal breathing from −8 mmHg at end‐expiration to approximately −15 mmHg at end‐inspiration (Miserocchi et al. 1990, 1991; Taylor and Parker 1985). On this basis, in thoracic tissues fluid continuously flows:

– from the systemic microvasculature of the intercostal spaces into the parietal subpleural interstitium (Moriondo et al. 2005), and from there into the pleural space;
– from the systemic capillaries of the abdominal parietal and visceral walls into the peritoneal space (Negrini et al. 1990, 1993), resulting in peritoneal fluid filtration;
– from the pulmonary capillaries, characterized by a relatively low hydraulic pressure of ~10 mmHg (Raj and Chen 1986; Negrini, Gonano, and Miserocchi 1992), into the surrounding interstitium, which exhibits extremely low *P*_int_ values (Miserocchi et al. 1990, 1991). In the lung, bulk fluid filtration occurs at both the arteriolar and venous sides of the pulmonary capillaries (Negrini, Gonano, and Miserocchi 1992), making lymphatic drainage the primary route for fluid and solute removal, at least under normal physiological conditions. Fluid filtration is accompanied by passive convective and diffusive fluxes of molecules spanning a wide range of molecular weights, depending on the sieving properties and the electrical charge of the endothelial layer, in accordance with pore theory (Patlak et al. 1963; Curry 1984; Adamson and Michel 1993; Rippe and Haraldsson 1994).

**FIGURE 1:**
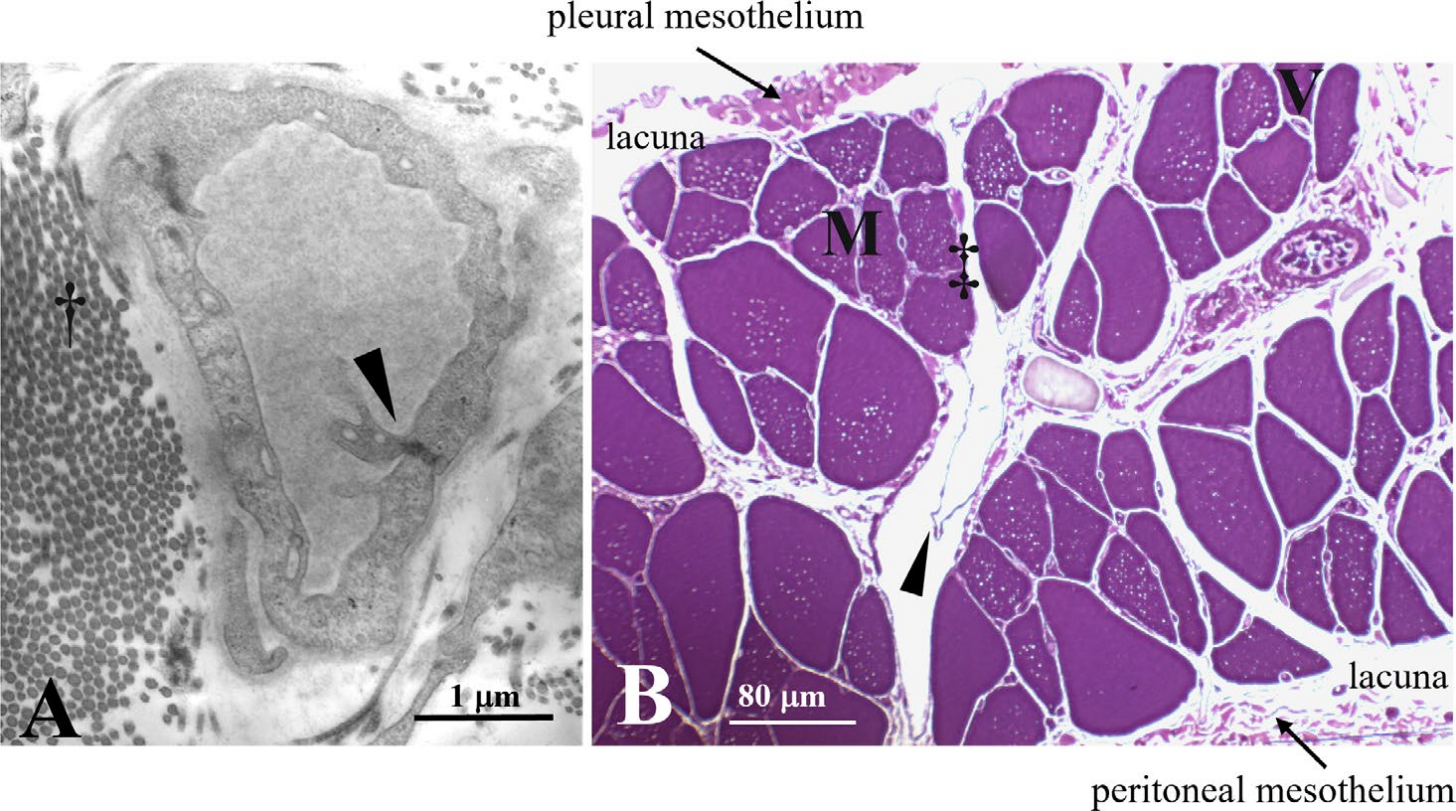
Details of the rat diaphragmatic lymphatic vasculature, showing primary, and secondary intraluminal valves. (A) Transmission electron microscopy (TEM) representative image of an initial lymphatic vessel lined by a single endothelial layer and surrounded by collagen bundles (†). A primary unidirectional valve (arrowhead) is visible in the vessel wall, formed by endothelial leaflets protruding into the lumen. (B) Semithin transverse section showing the subpleural (top) and subperitoneal (bottom) lymphatic lacunae, skeletal muscle fibers (M), erythrocytes filling blood vessels (V), and transverse lymphatic collectors lined by a discontinuous endothelium (‡). The arrowhead indicates a secondary intraluminal lymphatic valve.

To maintain constant tissue fluid volume and composition, the filtered fluid and solutes must be removed at an equivalent outflow rate through the lymphatic vasculature, which is organized into initial vessels (Figure 1A) and larger, more structured, collecting ducts (Figure 1B). Within this one‐way vascular circuit, lymph is conveyed unidirectionally and centripetally through secondary lymphoid organs, the lymph nodes, until it ultimately drains into the venous bloodstream.

### 2.1 Initial Lymphatics

Initial lymphatics, also termed lymphatic capillaries, are small, blind‐ended, saccular, finger‐like vessels, embedded within the interstitial tissue in close proximity to the fibers of the extracellular matrix scaffold (Figure 1A). Their diameter may vary from ~20 μm in the lung (Leak and Jamuar 1983) up to 100 μm in the diaphragm (Grimaldi et al. 2006), and they are often highly irregular even within the same tissue. The lumen is lined by a single layer of lymphatic endothelial cells (LECs) supported by a mostly discontinuous basement membrane, without fenestrations. Unlike what is observed in most blood capillaries, interendothelial lymphatic clefts are wide enough to allow the passage of large macromolecules and even cells (Grimaldi et al. 2006; Schmid‐Schönbein 1990).

In thoracic initial lymphatics, anchoring filaments of Collagen VII are of particular functional importance (Grimaldi et al. 2006). When the matrix fibers are stretched and/or the interstitial space swells because of increased vascular fluid extravasation, the anchoring filaments are pulled outwards, leading to the expansion of the vessel lumen and, consequently, to a decrease in intraluminal lymphatic pressure (*P*_lymph_). The outward traction, due to by anchoring filaments on the lymphatic wall prevents the vessel lumen from collapsing, as would be expected on the basis of the decreased transmural pressure (Δ*P*_TM_ = *P*_lymph_−*P*_int_) across the lymphatic wall. Instead, the vessel expands (Leak 1970). Although all initial lymphatics are equipped with anchoring filaments, their role becomes essential in highly moving tissues, such as the thoracic organs, which are continuously exposed to cyclic respiratory and cardiac swings. Indeed, the anchoring filaments ensure a rapid and reliable transmission of the mechanical forces elicited in the surrounding tissue to the vessel lumen, thereby allowing tissue displacement to be exploited as a mechanism for lymph formation and propulsion.

The wall of intercostal and diaphragmatic initial lymphatics is surrounded by a thin endothelial layer, supported by a discontinuous basement membrane, and is devoid of lymphatic muscle cells (LMCs), although scant actin‐like filaments have occasionally been observed within the endothelial cells (Grimaldi et al. 2006; Schmid‐Schönbein 1990). In intercostal skeletal muscle, small initial lymphatics may run along both arcade and transverse arterioles (Schmid‐Schönbein 1990; Lee et al. 2021).

### 2.2 Stomata and Lacunae

**FIGURE 2:**
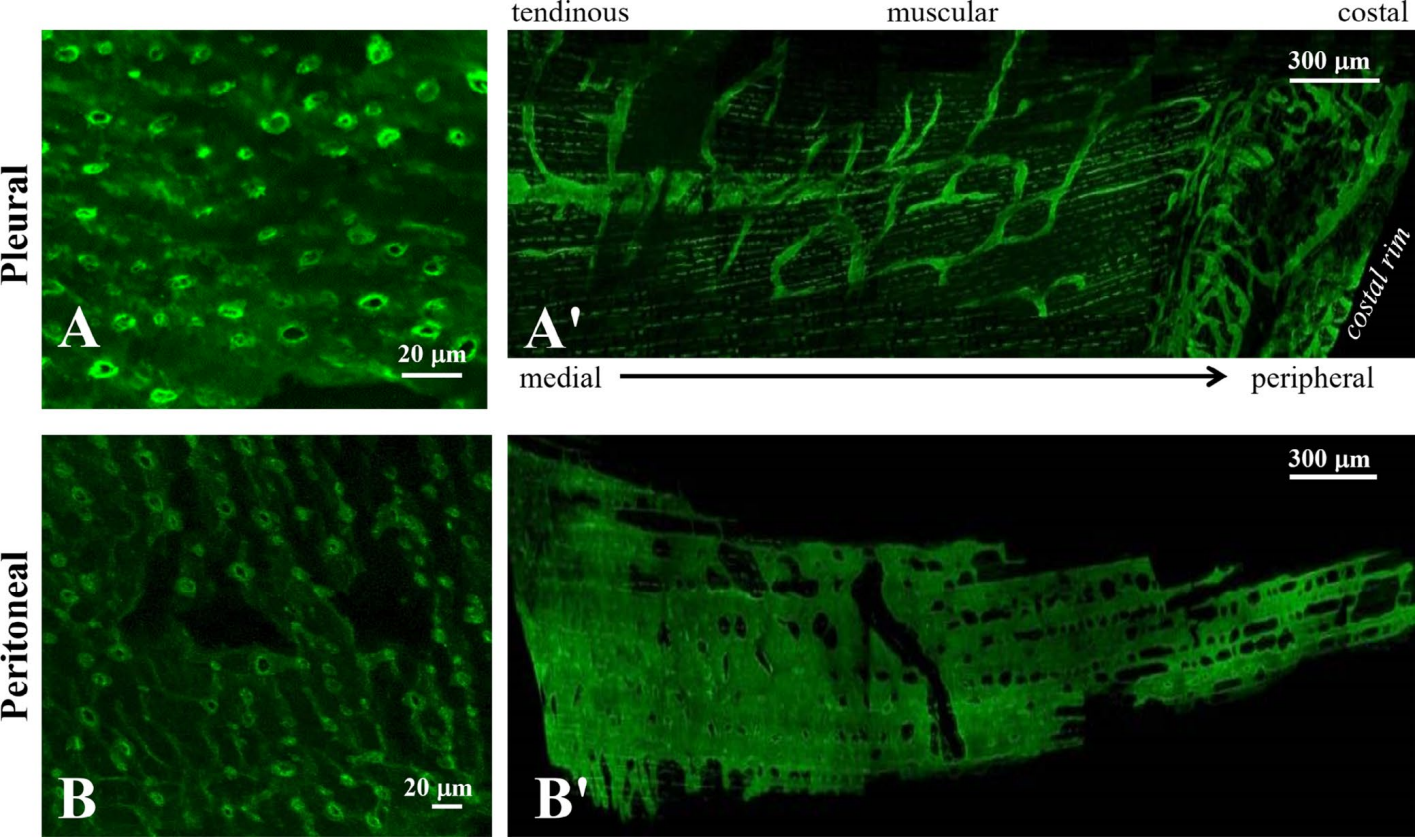
Diaphragmatic stomata and lacunae. Confocal images of mouse diaphragmatic pleural (A) and peritoneal (B) stomata, fluorescently stained with the lymphatic endothelial marker anti‐LYVE‐1 (lymphatic vessel endothelial hyaluronan receptor‐1). The underlying pleural (A') and peritoneal (B') lymphatic lacunae are organized in distinct submesothelial networks, spanning from the costal peripheral rim to the medial tendinous region of the diaphragm.

An important and unique feature exclusively encountered in the parietal (but not the visceral) pleura and in the peritoneum is the dissemination, on the mesothelial surface, of the so‐called “stomata” (Figure 2A,B), which are cylindrical‐like discontinuities that represent the origin of the lymphatic network from the pleural and peritoneal cavities (Wang 1975, 1985; Negrini et al. 1991; D. Negrini 2022). Although widely distributed over the costal (average density ~100/cm^2^) and the mediastinal parietal pleura, the highest density of stomata is encountered in the diaphragm (~8000/cm^2^), where they are distributed in the tendinous and muscular regions of both the pleural and peritoneal surfaces (Negrini et al. 1991; D. Negrini 2022). The stomata (average diameter ranges from ~0.5 to ~20 μm (Wang 1975; Wang 1985; Negrini et al. 1991; D. Negrini 2022)) empty into an extensive network of lymphatic submesothelial lacunae (D. Negrini 2022; Negrini, Del Fabbro, et al. 1992) located within the interstitial space beneath the mesothelial layer (Figure 2A',B'). No evidence of stomata has ever been reported in the visceral pleura, whose submesothelial interstitium is supplied by peripheral branches of pulmonary lymphatics that do not reach the mesothelial layer and do not connect directly with the pleural space (Albertine et al. 1982), so that the pleural and pulmonary lymphatic drainage are functionally independent of each other (Miserocchi et al. 1990).

Diaphragmatic stomata connect the pleural and peritoneal cavities with a system of extensive submesothelial lymphatic structures, usually defined as lacunae, thereby providing a direct anatomical pathway for fluid drainage (Negrini, Del Fabbro, et al. 1992). Confocal images of lymphatic lacunae stained with fluorescent anti‐LYVE‐1 (Lymphatic vessel endothelial hyaluronan receptor 1, a lymphatic endothelium marker) reveal a significantly different shape and spatial extent (Figure 2) on the pleural as compared with the peritoneal diaphragmatic side. Indeed, lacunae cover ~5% and ~30% of the pleural and peritoneal diaphragmatic surfaces, respectively, suggesting that they serve as high‐capacitance reservoirs able to cope with variable fluid drainage, which, even under physiological conditions, is much higher from the peritoneal than in from the pleural cavity (D. Negrini 2022).

On the pleural side (Figure 2A'), lacunae are organized in meshes of tubular vessels forming closed circuits, embedded within complex networks and connected by thinner interfibrillar vessels of variable size. Such structures are mostly distributed at the ventrolateral periphery of the diaphragm. On the peritoneal side (Figure 2B'), lacunae are instead organized as wide, flattened, lacy‐ladder‐shaped structures, with no evidence of lymphangions or tubular vessels. The lacunae wall is essentially devoid of lymphatic muscle cells (Grimaldi et al. 2006; Schmid‐Schönbein 1990; Leak 1970; Leak and Burke 1966) and they may therefore be considered specialized serosal initial lymphatics.

### 2.3 Unidirectional Lymphatic Valves

In all tissues, including the thoracic ones, bulk fluid flux into initial lymphatics takes place through primary unidirectional valves (Figure 1A) formed by overlapping cytoplasmic extensions of contiguous endothelial cells protruding into the lumen in close proximity to tight junctions (Grimaldi et al. 2006; Trzewik et al. 2001). In the diaphragm, primary valve openings may be up to 2–5 μm wide (Castenholz 1986, 1989), allowing the efficient transport of high molecular weight macromolecules and cells, a process that may be further facilitated by the high density of anionic sites on the surface of the valve leaflets. At present, it is not completely clear whether stomata entry is modulated by these one‐way valves. Diaphragmatic stomata, observed under a dual‐laser confocal microscope after anti‐LYVE‐1 staining, exhibit a variety of conformations, ranging from a circular or elliptical hollow shape to a partially closed or even completely occluded appearance (D. Negrini 2022). These observations may indicate either the existence of a gating structure at the stomata inlet and/or the coexistence of stomata at different stages of development. Further detailed investigations are required to clarify this issue. Once inside initial lymphatics, the newly formed lymph is driven along the entire lymphatic vasculature with the aid of the so‐called secondary intraluminal valves, consisting of two to three endothelial leaflets protruding into the vessel lumen, thereby forming a funnel‐like adjustable restriction (Castenholz 1989; Zawieja 2009; Davis et al. 2011). Secondary valves are essential because they partition the vessel into a sequence of adjacent, in‐series segments, the lymphangions, which represent the functional units of the lymphatic vasculature. Indeed, segmentation into a chain of lymphangions is essential for the development of local intraluminal *P*_lymph_ swings that drive both lymph formation and subsequent propulsion.

### 2.4 Collecting Vessels

The lacunae and the initial lymphatics empty into progressively larger ducts termed collecting vessels, (diameter > 175 μm, Figure 1B). Maculae adherens and occludens junctions are more frequent and tighter than in initial lymphatics, while the interendothelial gaps in the wall tend to be occluded (Grimaldi et al. 2006; Schmid‐Schönbein 1990; Leak and Burke 1966), thereby limiting the permeability of the wall to macromolecules and cells. A variable amount of elastic and collagen fibers is present in the basement membrane, which is surrounded by one or more layers of LMCs. Unlike what is observed in collecting lymphatics of other tissue districts, such as the mesenteric one described in detail in focused and elegant reviews (Zawieja 2009; Bridenbaugh et al. 2003; von der Weid and Zawieja 2004), in thoracic lymphatics the distribution of LMCs is far from being uniform, even within the same vessel or loop structure (Moriondo et al. 2013). LMCs may either spontaneously contract (Davis et al. 2024) or be triggered by external innervation via both cholinergic and adrenergic unmyelinated nerve fibers (Okiemy et al. 2003). In the diaphragm, collecting vessels are clearly visible under the transparent mesothelial surface, appearing as darker‐than‐background conduits delimited by faint white borders. These vessels converge into ducts located deeper within the diaphragm, which are not experimentally detectable from the surface, which carry the lymph toward the peritracheobronchial lymph nodes via lymphatic tributaries running along either the pulmonary ligament or the esophagus (Okiemy et al. 2003). Lymph from intercostal and mediastinal parietal pleura empties directly into the right lymphatic duct. Tributaries from the thoracic organs, including intercostal tissues, lungs, heart, and diaphragm, discharge mostly into the right and partially into the left thoracic duct (Riquet et al. 2002; Liu et al. 2006). The latter is the largest lymphatic vessel in the body and receives ~75% of the total lymph flow, mainly originating from the abdomen and lower extremities (Cha and Sirijintakarn 1976).

## 3 Mechanisms of Lymph Formation and Propulsion in Thoracic Tissues

Transport of fluid and solute from the interstitial space to the lymphatic lumen occurs by bulk flow through the highly permeable unidirectional primary valves of the initial lymphatics (Grimaldi et al. 2006; Schmid‐Schönbein 1990; Castenholz 1989; Moriondo, Grimaldi, et al. 2007), which allow the free entry of fluid, interstitial hydrophilic molecules of any size, as well as cells, viruses, and bacteria. Since the endothelium of initial lymphatics offers no effective sieving to large macromolecules or even cells, the solute and protein concentration are essentially the same in the newly formed lymph and in the interstitial fluid, so that lymph flow (*J*_lymph_) is independent of the colloidosmotic pressure gradients between the lymphatic lumen and the surrounding interstitium (Curry 1984; Guyton et al. 1975). Therefore, *J*_lymph_ into lymphatic vessels is sustained exclusively by the difference between local *P*_int_ and *P*_lymph_ in initial lymphatics, according to Equation (2):

(2) $$J_{\text{lymph}}=K_{\text{lymph}}\cdot \left(P_{\text{lymph}}-P_{\mathrm{int}}\right)=K_{\text{lymph}}\cdot \Delta P_{\mathrm{TM}}$$

where *K*_lymph_ is the permeability of the lymphatic wall to water (Granger et al. 1984) and Δ*P*_TM_ is the transmural hydraulic pressure gradient that drives fluid entry into initial lymphatics. According to Equation (2), lymph formation occurs only when *P*_lymph_ drops below *P*_int_, so that Δ*P*_TM_ results negative. Importantly, initial lymphatics are delimited by a non‐elastic, collapsible wall that does not occlude because of the external stretch exerted by the anchoring filaments, even when exposed to a negative Δ*P*_TM_. (Grimaldi et al. 2006; Trzewik et al. 2001; Chen et al. 1997).

In Equation (2), *P*_lymph_ is the key parameter in both lymph formation and propulsion. Indeed, it is directly implicated in the two essential steps of lymph flow dynamics. The first step consists of driving fluid and solutes from the interstitial space or serosal cavity into the initial lymphatics, a process leading to lymph formation. This phase is particularly intriguing, as it requires *P*_lymph_ to become lower than *P*_int_, which in thoracic tissues may be significantly lower than atmospheric, particularly in the lung parenchyma (Miserocchi et al. 1990, 1991). Once fluid has entered the initial lymphatics, the newly formed lymph needs to be propelled forward along the collecting vessels, overcoming the resistance offered by viscous flow along the larger ducts and by percolation through lymph nodes, until it finally discharges into the venous bloodstream. At this stage, venous pressure at outflow sites of the major right and left collecting ducts is slightly positive, so that lymph driven from thoracic cavities must flow against a pressure gradient, being *P*_lymph_ in initial lymphatics always lower than central venous pressure. This peculiar flow dynamic is another major difference between lymphatic and blood circulation, which occurs only along, and not against, a pressure gradient (Solari et al. 2022).

Hence, on the one hand *P*_lymph_ in initial lymphatics must be lower, often markedly subatmospheric, than the surrounding *P*_int_ and, on the other, the same *P*_lymph_ value must progressively increase along the lymphatic network until it overcomes central venous pressure. To meet this dual requirement and attain the appropriate intraluminal *P*_lymph_ value, lymphatic vessels exploit a tissue‐tailored combination of two different but cooperative mechanisms, defined as “intrinsic” and “extrinsic.”

### 3.1 The Intrinsic Mechanism and the Role of Lymphatic Muscle Contraction and Relaxation Phases

The contractile machinery, elegantly described elsewhere (Zawieja 2009; von der Weid and Zawieja 2004; Kirkpatrick and McHale 1977), relies on the coordinated interaction between the contraction–relaxation cycle of LMCs and the behavior of intraluminal unidirectional lymphatic valves (secondary valves, as defined above), which passively open and close according to the hydraulic transvalve pressure gradient (Davis et al. 2011; Bridenbaugh et al. 2003; von der Weid and Zawieja 2004; Bertram and Davis 2023; Davis and Zawieja 2024).

In the absence of a central pump, such as the heart in the blood circulatory system, when LMCs in the wall of a given lymphangion contract, a series of events ensues. Locally, in the contracted lymphangion, *P*_lymph_ increases, overcoming the outflow pressure gradient with respect to the adjacent lymphangion; therefore, fluid may move both upstream and downstream along the resulting *P*_lymph_ gradient. Since secondary valves are structurally designed as funnel‐like unidirectional tunnels (Figure 1B), in most cases potential backflow occludes the valve leaflets, thereby closing the valve itself, while in other cases the valve remains biased in the open position, allowing some lymph backflow (Davis et al. 2011; Solari et al. 2026). Lymph that progresses through the valve to the adjacent, relaxed, lymphangion is then driven centripetally, while the previously contracted lymphangion subsequently relaxes, and its intraluminal *P*_lymph_ suddenly drops, favoring, upstream up to the initial lymphatics, the entry of interstitial fluid and the formation of new lymph. This contraction–relaxation cycle repeats at a frequency of about 19–20 cycles/min (Moriondo et al. 2016; Solari et al. 2017), starting within the smallest vessels and propagating as a contractile wave from one lymphangion to the next, driving the lymph throughout the entire lymphatic vasculature. Notably, even though the lymph apparently flows against a tissue‐to‐venous hydraulic pressure gradient, because of the segmentation of the vasculature into adjacent lymphangions and the asynchronous contraction of LMCs, net flow always occurs downstream from distal toward proximal lymphangions (Hald et al. 2018). The same mechanism is involved in lymph formation. Indeed, LMCs relaxation is accompanied by lymphangion chamber enlargement and local *P*_lymph_ drop. When *P*_lymph_ falls below the surrounding interstitial *P*_int_, the intrinsic mechanism alone is sufficient to sustain simultaneously both lymph formation and subsequent propulsion. This situation occurs in still tissues, such as the mesentery, where the alternation of LMCs contraction and relaxation phases is the only source of the *P*_*lymph*_ waves needed to support lymph formation and progression.

Contraction of LMCs may occur either spontaneously or be triggered by external stimuli. Because of the experimental advantages offered by these tissue models, the properties of active LMCs have been analyzed primarily in collecting ducts or in mesenteric lymphatics (Zawieja 2009; von der Weid and Zawieja 2004; Kirkpatrick and McHale 1977), According to these studies, contraction is triggered by activation of specific, spontaneously firing pacemaker sites, from which the contractile wave is transmitted throughout the entire network. It has been demonstrated that LMCs contractions immediately follow bursts of small transient depolarizations (STDs) of the cell membrane, sustained by spontaneous transient inward currents (Kirkpatrick and McHale 1977; Allen et al. 1983; Van Helden 1993; von der Weid 1998). It has also been proposed that LMCs depolarization requires the involvement of voltage‐gated L‐type Ca^2+^ channels (Kirkpatrick and McHale 1977; Allen et al. 1983; Telinius et al. 2014) and/or voltage‐gated Na^+^ channels (Telinius et al. 2015), as sources of action potentials and the subsequent propagation of the LMCs contractile wave, in a manner similar to that observed in skeletal muscle. (Trzewik et al. 2001; Skalak et al. 1984).

**FIGURE 3:**
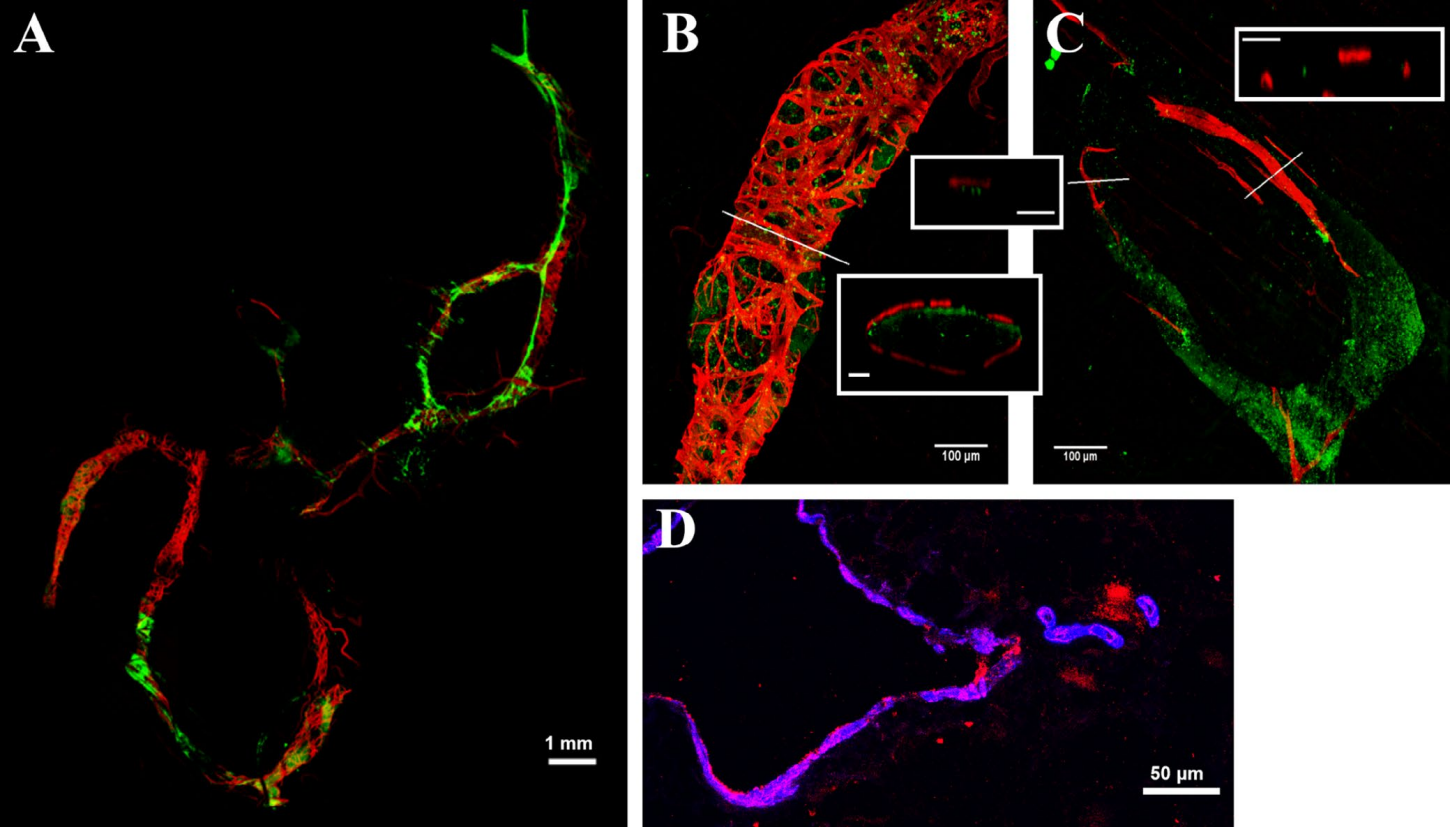
Distribution of lymphatic muscle within the wall of thoracic lymphatic vessels. (A) Whole‐mount image of rat diaphragmatic lymphatic loops, stained with FITC‐dextran (green) and anti‐SMCα (red), highlighting the vessel lumen and the lymphatic muscle mesh, respectively. The LMCs network surrounding the vessel wall is not homogeneously distributed along the vessel perimeter, as evident in higher‐magnification images. (B) Representative image of a spontaneously contracting site, whereas (C) shows an inert site. Transverse cross‐sections obtained at the positions indicated by white lines are also shown (inset scalebar 25 μm). (D) Diaphragmatic lymphatic sections stained with anti‐HCN4 channel (red) and anti‐SMCα (blue), highlighting HCN4 localization within the lymphatic muscle layer in the wall of a spontaneously contracting vessel (scalebar 50 μm).

**FIGURE 4:**
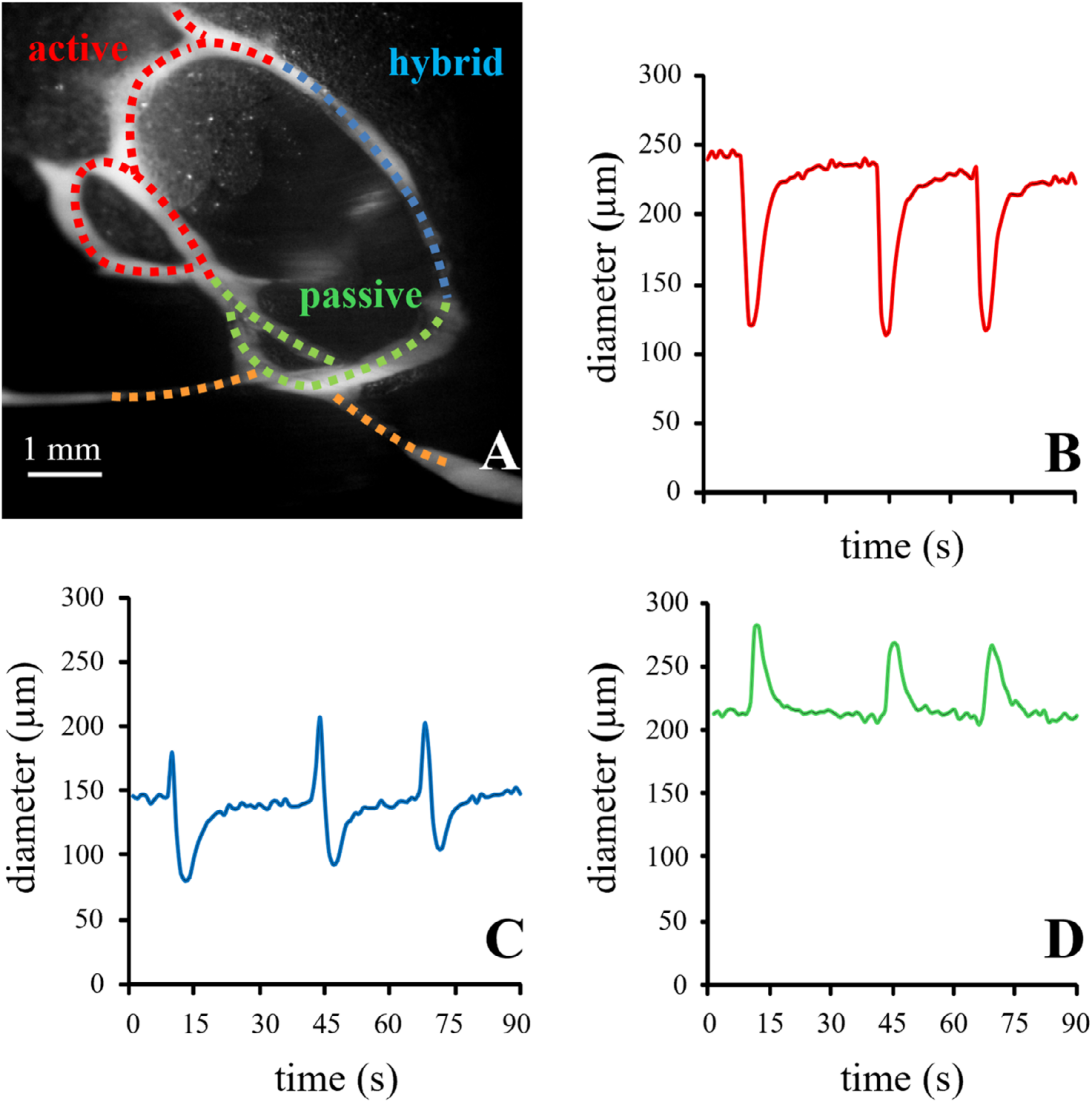
Segmental functional variability in diaphragmatic lymphatic loops. Simultaneous changes in vessel diameter recorded in adjacent segments within the same rat diaphragmatic lymphatic loop. In the upper‐left portion of the loop in panel A (red dotted line) the vessel spontaneously contracts, and its diameter (panel B) rapidly decreases after a relaxation phase, indicating an «active» contractile behavior. In the segment adjacent to the active tract (A, blue dotted line), the diameter suddenly decreases (C) only after a rapid enlargement, suggesting a «hybrid», stretch‐activated, contractile behavior, likely triggered by the arrival of fluid from the adjacent actively contracting site. In the lower segment of the loop (green dotted line), no active contractile activity can be detected, and the vessel diameter (D) passively distends when fluid enters the segment. The yellow line in panel A identifies a lymphatic segment in which the diameter does not show any significant change over time (inert site).

Compared with what is commonly observed in lymphatic districts supplying soft, relatively immobile tissues (Bridenbaugh et al. 2003; Davis et al. 2024; Kirkpatrick and McHale 1977) LMCs and alpha‐smooth muscle actin (SMCα) are extremely sparsely distributed in thoracic lymphatics (Moriondo et al. 2005) (Figure 3). Nonetheless, multiple evidence indicates that thoracic lymphatics possess the cellular machinery required to exploit the intrinsic mechanism. Indeed, spontaneous *P*_lymph_ waves have been recorded in in situ diaphragmatic peripheral loops, both in open and closed chest (Negrini and Del Fabbro 1999; Negrini et al. 2004). Moreover, in spontaneously breathing anesthetized rodents, intrapleural injection of amiloride, a well‐known inhibitor of Na^+^‐Ca^2+^ exchangers, which are possibly involved in the intrinsic mechanism, reduces pleural lymphatic egress rate by ~40% (Negrini et al. 1994) and segments of lymphatic loops belonging to isolated rodent diaphragm specimens (Figure 4A) may spontaneously contract at a relatively constant rate (Moriondo et al. 2013, 2008). Direct exposure of spontaneously contracting lymphatic vessels located in isolated peripheral diaphragm specimens to selective and nonselective inhibitors of the hyperpolarization‐activated cyclic nucleotide‐gated (HCN) channels, such as cesium chloride (CsCl), ivabradine, and ZD‐7288, demonstrated a direct dependence of lymphatic contractile frequency on HCN channels activity in this body area (Negrini et al. 2016). In particular, the selective HCN channel blocker ivabradine completely inhibits contractility in most of the analyzed vessels, strongly suggesting that these channels, expressed in thoracic lymphatics (Figure 3D), are responsible for the spontaneous triggering of LMCs contraction. HCN channels belong to a family of voltage‐gated ion channels that, in diaphragmatic lymphatics, are expressed in four different isoforms (HCN1‐4) clustered at selected sites of the lymphatic muscle within the vessel wall. The involvement of HCN channels in spontaneous membrane depolarization, and therefore in cellular pacing, has been demonstrated in thalamic neurons (Biel et al. 2009) as well as in the heart. (DiFrancesco 1982; DiFrancesco et al. 1991). In particular, in cardiac sinoatrial myocytes, HCN channels are responsible for the spontaneous Na^+^‐dependent depolarization that triggers the automatic firing of sinoatrial node myocytes (DiFrancesco et al. 1991; DiFrancesco 1981), thereby setting heart rate. Therefore, although operating over very different frequency ranges, transmembrane ion channels belonging to the same HCN channel family trigger spontaneous contractions in both cardiac myocytes and lymphatic muscle.

#### 3.1.1 Role of Valves in Intrinsic Mechanism of Thoracic Lymphatics

In a complex, branched, lymphatic network composed of vessels with an elliptical cross‐sectional geometry, such as the diaphragmatic ones, intraluminal valves may play an important auxiliary role. The *P*_lymph_ gradient needed to open a valve and overcome its hydraulic resistance is ~1.5 mmHg, a value consistent with those observed in extrathoracic lymphatics (Davis et al. 2011, 2024; Solari et al. 2026), but much lower than the *P*_lymph_ swings elicited by extrinsic contractions in diaphragmatic and intercostal lymphatics (Moriondo et al. 2005, 2015). Intraluminal flow velocity, obtained by ex vivo fluorescent microsphere tracking within the vessel lumen of diaphragmatic lymphatics, indicates that lymph velocity is constant in segments without valves, but significantly increases when flowing through the narrower functional orifice of an open valve compared with nonvalved segments, as already previously documented (Pujari et al. 2020). However, in these vessels, which are also highly branched, lymph velocity tends to be lower than that observed in extrathoracic vessels, such as mesenteric lymphatics (Rousselle et al. 1997; Guyton et al. 1971). It has been reported that, when flow is driven exclusively by the intrinsic mechanism and the transvalve *P*_lymph_ gradient is unfavorable to forward progression, intraluminal valves do not occlude completely, allowing some fluid backflow, which therefore occurs, upon lymphangion wall contraction, both in segments without valves and in those equipped with valves. Nevertheless, since backflow is consistently smaller in valved than in nonvalved segments, the presence of valves provides a significant advantage in terms of net fluid progression, even if they remain biased toward an open state (Contarino and Toro 2018). These observations suggest that the intrinsic mechanism can be effectively exploited also in thoracic tissues, despite the dominant mechanical support provided by the surrounding contracting skeletal muscle to lymphatic vessels. Conversely, since lymph backflow does not occur in diaphragmatic vessels exposed to cyclic tissue motion (Moriondo et al. 2015), these findings confirm that the intrinsic mechanism alone cannot, by itself, sustain adequate lymph drainage and propulsion, which therefore necessarily require the contribution an of extrinsic mechanisms (Moriondo et al. 2016).

### 3.2 The Extrinsic, Tissue Motion‐Dependent Mechanisms

**FIGURE 5:**
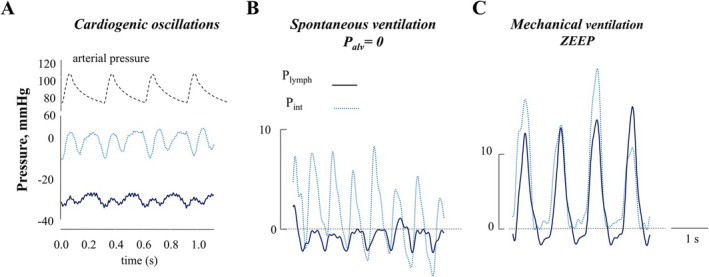
Impact of cardiac and respiratory cycles on interstitial and lymphatic pressure. (A) Cardiogenic oscillations in diaphragmatic interstitial pressure (*P*_int_, dotted line) and intraluminal hydraulic lymphatic pressure (*P*_lymph_, solid line) recordings, occurring nearly in phase with arterial systemic pressure oscillations (dashed line). (B) *P*_int_, and *P*_lymph_ profiles recorded in the submesothelial intercostal interstitium and lymphatic vessels, respectively, during spontaneous tidal breathing. (C) *P*_int_, and *P*_lymph_ profiles recorded in the submesothelial intercostal interstitium and lymphatic vessels, respectively, during mechanical ventilation performed at zero end‐expiratory pressure (ZEEP) and with a tidal volume equal to that of spontaneous breathing.

In contrast to what is observed in mesenteric or subcutaneous lymphatic districts, where *P*_int_ is close to atmospheric and *P*_lymph_ oscillations depend exclusively upon LMCs contraction–relaxation waves, all thoracic lymphatics experience large *P*_lymph_ and Δ*P*_tm_ swings, elicited by tissue displacement and/or stresses associated with cardiac (Figure 5A) and respiratory cycles (Figure 5B,C) (Moriondo, Grimaldi, et al. 2007; Negrini et al. 2004).

#### 3.2.1 Effect of Cardiac Cycle

The alternation between cardiac systole and diastole has a direct impact, both in terms of mechanical forces and stresses, as well as stretch and compression exerted on solid matrix fibers, not only on atrial and ventricular myocardium, but also on the lung, intercostal tissues, the diaphragm, pleural fluid, and, to a much smaller extent, the subdiaphragmatic peritoneal regions. Indeed, cardiac activity is accompanied by (a) displacement of mediastinal tissues, lung parenchyma, and the diaphragmatic phrenic center, and (b) by propagation of aortic and pulmonary arterial pulse waves along major thoracic and extrathoracic vessels. The impact of these local events results in rhythmic cardiogenic oscillations that can be observed, at the same frequency as the heartbeat, in *P*_int_ and *P*_lymph_ recordings (Figure 5A) in all thoracic organs where they have been directly measured (Moriondo et al. 2005; Moriondo, Grimaldi, et al. 2007; Negrini et al. 2004). On average, cardiac motion is accompanied by *P*_int_ and *P*_lymph_ swings of ~5 mmHg (Moriondo, Grimaldi, et al. 2007; Negrini et al. 2004). In intercostal subpleural lymphatics, *P*_int_ and *P*_lymph_ may oscillate either in phase or out of phase with each other, determining the development of local Δ*P*_tm_ values on the order of ~4 mmHg (Moriondo et al. 2005), a value comparable to that observed across the wall of diaphragmatic lymphatics. Accordingly, in intercostal and diaphragmatic interstitial tissues, cardiogenic oscillation alone, by supporting the occurrence of a positive Δ*P*_tm_, may be sufficient to sustain local lymph formation and propulsion.

Comparable estimates cannot currently be derived for pulmonary and cardiac lymphatics, whose *P*_lymph_ values have not yet been measured because of technical experimental complexities. Nevertheless, one may reasonably expect the cardiogenic effect to be even more pronounced in these organs. By contrast, the cardiac cycle exerts a negligible effect on pleural and peritoneal fluid pressures, in which no detectable cardiogenic swings have been observed (Miserocchi et al. 1988; Negrini and Miserocchi 1989).

#### 3.2.2 Mechanical Impact of Tidal Breathing on Thoracic Interstitial Spaces and Lymphatics

The recurrent inspiratory–expiratory sequence profoundly affects lymphatic drainage in thoracic tissues, in a much more complex and physiologically relevant manner than that described for the cardiac cycle. The most comprehensive analysis of events occurring along the wall of lymphatic vessels during spontaneous tidal breathing has been obtained in intercostal tissues, where it was possible to directly record *P*_int_ and *P*_lymph_ in the interstitium and in lymphatic vessels lying immediately below the parietal mesothelium, respectively (Figure 5B). During inspiration, contraction of the external intercostal muscles exerts a tangential stress on parasternal or subpleural paracostal interstitial fibers. The inspiratory rib displacement, and the associated stress exerted on interstitial structures, are greater in rostral than in caudal intercostal spaces, causing heterogeneous tangential stresses distributed along the rostrocaudal axis of the thorax (De Troyer et al. 1999; De Troyer and Wilson 2002). A second factor to consider is the additional decrease in pleural liquid pressure toward more negative values during inspiration, which, compared with end‐expiration determines a stronger orthogonal inward traction applied to the pleural surface. As a consequence of this complex mechanical interplay:

- at end‐expiration, average *P*_lymph_ (~−2.5 mmHg) is always significantly lower than mean *P*_int_ (~ 3 mmHg), thereby favoring a net fluid flux into initial lymphatics;
- during spontaneous inspiration, both *P*_lymph_ and *P*_int_ significantly decrease, to ~−20 mmHg and ~−12 mmHg, respectively, further increasing Δ*P*_tm_ and thereby enhancing lymph formation. In fact, because of inspiratory mechanical changes, *P*_int_ markedly decreases during inspiration, and this *P*_*int*_ drop is efficiently transmitted to the compliant wall of initial lymphatics (Schmid‐Schönbein 1990) through the anchoring filaments that connect the vessel wall to neighboring matrix fibers.

On average, over the entire cycle Δ*P*_tm_ is ~ −7 mmHg, although it oscillates within a single breath. Notably, mechanical ventilation (Figure 5C), performed at the same tidal volume and respiratory rate as spontaneous breathing, abolishes Δ*P*_TM_ suggesting that lymph formation and propulsion depend on stresses elicited by active intercostal muscle contraction, rather than simply on the passive enlargement and collapse of the chest. On this basis, one may expect that patients undergoing mechanical ventilation, even at zero end‐expiratory alveolar pressure (ZEEP), may be at increased risk of developing clinically relevant pleural effusions, as a consequence of markedly reduced pleural lymphatic drainage.

## 4 Organization and Role of the Diaphragmatic Lymphatic Vasculature

The organization of the diaphragmatic lymphatic network is highly complex. On both the pleural and peritoneal sides, submesothelial lacunae, embedded in connective tissue predominantly composed of collagen (Grimaldi et al. 2006), empty into a network of superficial and deep lymphatic collectors arranged both in parallel and orthogonal with respect to muscular and/or tendinous fibers (Grimaldi et al. 2006; Moriondo, Grimaldi, et al. 2007). Depending on their location within the diaphragm, lymphatic vessels may be: (a) very superficial, delimited by the pleural or peritoneal mesothelium, and partially surrounded by submesothelial tissue; (b) located deeper within the diaphragm, surrounded by submesothelial tissue and encircled by skeletal muscle or tendinous tissue; and (c) positioned even deeper, in the central thickness of the diaphragm, and entirely encompassed by the muscular or tendinous tissues. These vessels differ not only in their anatomical position in the diaphragm but, more importantly, in the response of their wall to local mechanical stresses. Indeed, their behavior during the diaphragmatic contraction–relaxation cycle, and the associated elongation and shortening of matrix fibers, is strongly influenced by the stiffness of the vessel wall, the mechanical properties of the adjacent interstitium, and the fraction of the vessel surface area interfacing with compliant versus stiff tissues, respectively (Figure 5).

**FIGURE 6:**
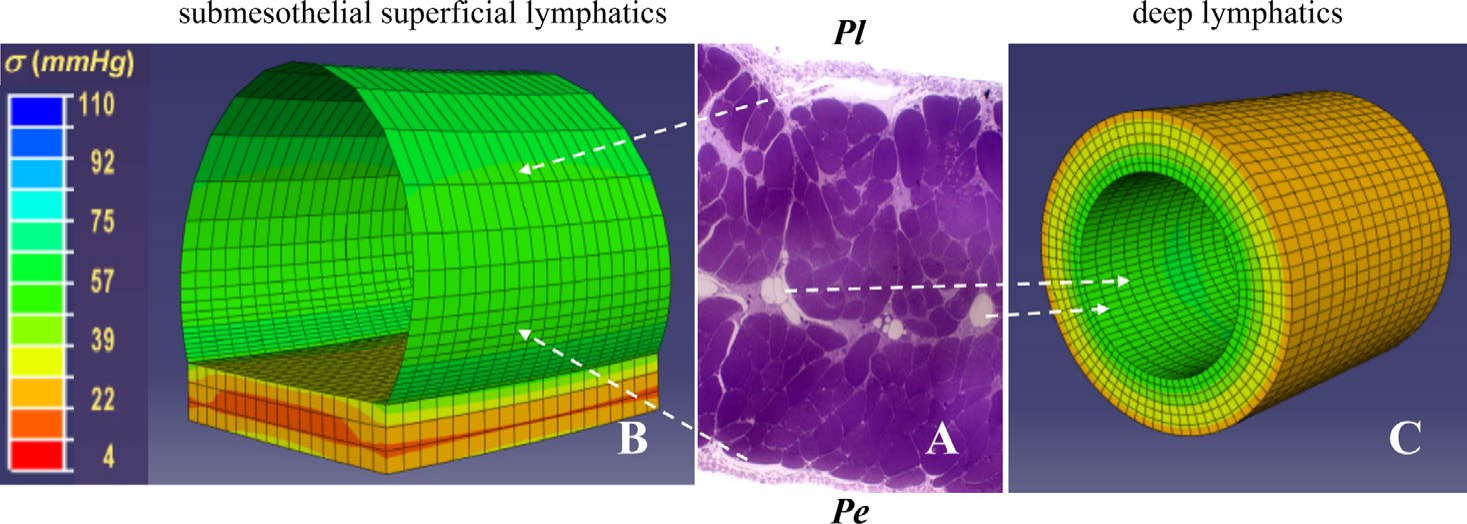
Mechanical behavior of diaphragmatic lymphatic vessels. Lymphatic vessels may be located either superficially or deeply within diaphragmatic tissue, as illustrated in panel A. Finite element modeling of circumferential stress distribution in the wall of an ideal diaphragmatic lymphatic vessel indicates that circumferential stress (color scale ranging from red—low stress—to blue—high stress) is higher in the wall of the superficial submesothelial vessel (B), located beneath the pleural or peritoneal mesothelium, which undergoes the largest deformation when exposed to pressure changes. By contrast, the deeper vessel (C), surrounded by skeletal muscle fibers and characterized by greater wall stiffness than the submesothelial vessel, experiences lower and homogeneous wall stress, particularly during diaphragmatic contraction. Accordingly, superficial submesothelial vessels are functionally suited for fluid absorption from serosal spaces, whereas deeper lymphatics are primarily involved in lymph propulsion.

Direct measurements performed in the rat diaphragm (Moriondo et al. 2010) showed that the elastic modulus of skeletal muscle tissue is much higher than that of the lymphatic mesothelium or the lymphatic endothelium alone. Based on these parameters, Finite Element modeling of vessel mechanical performance revealed that the compliance of superficial submesothelial lymphatic vessels is approximately two orders of magnitude greater than that of deeper, markedly stiffer, lymphatics. From a functional standpoint, this implies that, for a given tissue stress, widening of interendothelial gaps in the lymphatic wall would be more pronounced in vessels with a more compliant wall (Figure 6A), such as initial submesothelial vessels, which are rich in primary valves (Grimaldi et al. 2006; Trzewik et al. 2001), as well as in mesothelial stomata of the pleural and peritoneal surfaces. Accordingly, stomata and lacunae exhibit both morphological and mechanical features that enable them to function as specialized sites of lymph formation.

**FIGURE 7:**
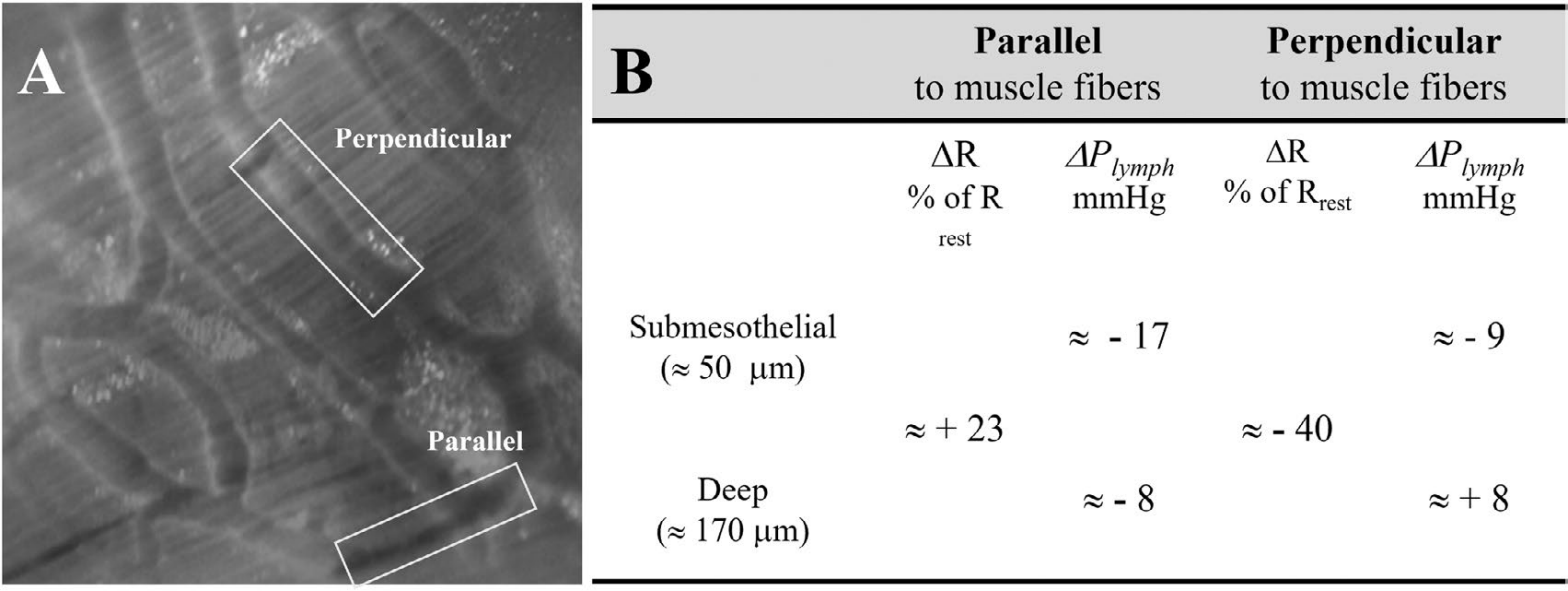
Impact of the relative alignment of lymphatic vessels and skeletal muscle fibers. (A) Representative image showing lymphatic vessels oriented either parallel or perpendicular to the underlying skeletal muscle fibers in the rat diaphragm. (B) Extrinsically induced changes in vessel radius (ΔR, expressed as a percentage of the corresponding resting value, R) and intraluminal pressure (Δ*P*_lymph_) in parallel‐ and perpendicular‐oriented lymphatic vessels located at different depths within the diaphragmatic tissue.

By contrast, vessels partially or, even more so, completely embedded in muscular or tendinous fibers display a more rigid wall, through which tissue forces generated by the respiratory and cardiac cycles are more uniformly transmitted to the vessel wall and internal lumen, thereby rendering forward fluid propulsion more efficient in these vessels than in the more superficial ones (Figure 6C). In addition to their location within the diaphragmatic thickness, a further factor that influences local lymphatic function is the orientation of lymphatic vessels with respect to adjacent skeletal muscle fibers. The alignment of diaphragmatic lymphatics with respect to the radial arrangement of muscular fibers is anatomically highly variable, making such an analysis intrinsically complex. However, a simplified approach, restricting the assessment of the effect of skeletal fiber contraction on lymphatic vessels aligned either parallel or orthogonal (Moriondo et al. 2015) to the major muscular fiber axis (Figure 7A,B) revealed that:

- in vessels oriented perpendicular to muscle fibers and lying deep within diaphragmatic tissue, skeletal muscle contraction reduces vessel diameter while simultaneously increasing local *P*_lymph_, whereas the opposite effect is observed in more superficial vessels, where muscle contraction causes vessel enlargement and a concomitant decrease in *P*_lymph_;
- conversely, in vessels aligned parallel to the major axis of muscle fibers, muscle contraction is always accompanied by an enlargement of the vessel diameter and a decrease in *P*_lymph_, independently of vessel depth within the tissue

Overall, a key message of this pivotal study (Moriondo et al. 2015) is that the respiratory cycle, and the associated contraction–relaxation alternation that accompanies it, does not exert a homogeneous effect on local lymphatic function but, rather, may simultaneously enhance lymph propulsion in some vessels and lymph formation or redistribution in others within the same lymphatic network, depending on vessel orientation and depth within the diaphragm. Consistent with these observations, and given that diaphragmatic lymphatics provide drainage of both pleural and peritoneal fluid (Negrini et al. 1990, 1993; Moriondo, Grimaldi, et al. 2007), contraction of skeletal muscle fibers of the ventromedial diaphragmatic dome appears to be essential to drive serosal fluid from submesothelial lymphatic structures into deep, parallel‐oriented vessels during muscle contraction, and subsequently into deep, perpendicularly oriented ducts, which are preferentially involved in propelling lymph forward along the circuit and, ultimately, out of the diaphragm.

Accordingly, in the diaphragm, factors such as the geometric organization of the lymphatic network with respect to muscular fibers and cyclic motor unit recruitment appear to be effectively integrated to provide the most efficient mechanical and fluid‐dynamic support to the lymphatic vasculature, thereby optimizing both lymph formation and propulsion.

### 4.1 Cooperation Between Intrinsic and Extrinsic Mechanisms in the Diaphragm

Despite the prevailing role of the extrinsic mechanism in sustaining diaphragmatic lymphatic function, and notwithstanding the evidence that LMCs are not homogeneously organized within the vessel wall, lymphatic vessels characterized by spontaneously contracting regions have been documented (Moriondo et al. 2013). In particular, a mesh of lymphatic muscle can be found in the wall of vessels organized in peripheral loops, which are usually, but not necessarily, partitioned into lymphangions and often arranged to form circular or oval rings that may connect either to another loop or to linear ducts, thereby forming a highly complex circuit. The role played by loop structures in the diaphragmatic lymphatic network is multifaceted and still not completely understood. Indeed, within a single loop (Figure 4A), spontaneously contracting sites (Figure 4B) may coexist with: (a) passive tracts (Figure 4D) that distend when fluid enters and tend to collapse when fluid leaves the segment, without showing intrinsic contractile activity; (b) stretch‐activated portions (Figure 4C) in which contraction is triggered by vessel enlargement upon fluid entry; and (c) inert segments of the loop (not shown in Figure 4), whose diameter appears completely unaffected by the behavior of adjacent segments (Moriondo et al. 2013). Most loops lie over the pleural muscular diaphragm, close to the external peripheral rim. In this region, known as the “apposition zone,” the diaphragm directly faces the thoracic wall, from which it is divided by a pool of pleural fluid. Because of its large radius of curvature, in the apposition zone the craniocaudal displacements of the diaphragm, as well as the tissue stresses elicited by inspiratory muscle contraction, are reduced compared with more medial diaphragmatic regions and may therefore be insufficient to support lymphatic function. Under these conditions, the presence of lymphatic loop segments capable of intrinsic contraction may significantly contribute to supporting and coordinating lymphatic flow in this peripheral, relatively motionless diaphragmatic area. Table 1 summarizes the key parameters of lymph flow in the diaphragmatic lymphatic network, driven by both extrinsic and intrinsic mechanisms.

**TABLE 1:** Comparison of parameters describing flow dynamics sustained by the intrinsic and extrinsic mechanisms in lymphatic vessels supplying the peripheral diaphragm in the rat.

	Extrinsic	Intrinsic
Net lymph progression/contraction, μm	441.9 ± 159.2	14.1 ± 2.9^a^
Average wall shear stress, dyne/cm	0.23 ± 0.08	0.02 ± 0.01^a^
Wall shear stress range, dyne/cm	0.02–0.55	0.001–0.05
Reynold's number	9.6 × 10^−2^	8.1 × 10^−3^
Stroke volume (SV), pL	458 ± 172	244 ± 91^a^
Ejection fraction (EF), %	54 ± 3	27 ± 3^a^
Computed net lymph flow, nL/min	39.8 ± 11.4	4.0 ± 1.4^a^
Frequency, cycles/min	46.7 ± 1.3^b^	20.6 ± 1.5^a^

*Note:* Stroke volume (SV) was computed for an ideal lymphatic segment 105.5 μm in length as SV = EDV‐ESV, where EDV and ESV are the end‐diastolic and end‐systolic volumes, respectively. Ejection fraction (EF) was calculated by analogy with the cardiac cycle as EF = SV/EDV. Data are from ref. (Moriondo et al. 2016). Values are expressed as mean ± SE.

^a^ Significantly different from extrinsic; paired *t*‐test, *p* < 0.05.

^b^ Spontaneous respiratory rate (breaths/min) recorded in adult, healthy, anesthetized rats. Data are from ref. (Moriondo et al. 2012).

## 5 Lymphatic Drainage of Pleural and Peritoneal Cavities

The mammalian pleural and peritoneal spaces are fluid‐containing cavities of mesodermal origin, anatomically separated by the diaphragm, whose lymphatic vasculature plays a critical role in the maintenance of physiological fluid homeostasis in both cavities. The parietal pleural mesothelium lines the inner surface of the thorax, the pleural diaphragmatic dome, and the mediastinal tissues, while the visceral mesothelium covers the entire lung surface and its fissures. Similarly, the parietal peritoneal mesothelium lines the inner abdominal wall, whereas the visceral one covers the abdominal organs. Both spaces contain extracellular fluid with a variable content of plasma proteins (Negrini et al. 1990, 1993), which is passively filtered: (a) into the pleural space across the parietal pleura, but not across the visceral one, which is supplied by the low‐pressure pulmonary circulation (Raj and Chen 1986; Negrini, Gonano, and Miserocchi 1992) and (b) into the peritoneal space through the parietal and visceral peritoneum, which are supplied by the systemic circulation. To ensure efficient and nearly frictionless sliding of thoracic and abdominal organs within their respective cavities, pleural and peritoneal fluid turnover must be optimized, a function almost entirely ensured by lymphatic drainage of the two cavities. To this end, the parietal, but not visceral, pleura and both the parietal and visceral peritoneum are supplied by an extensive mesh of lymphatic stomata and lacunae, which are characteristic features of serosal mesothelia, and are far more densely distributed on the peritoneal than on the pleural layers (Negrini et al. 1991; D. Negrini 2022; Negrini, Del Fabbro, et al. 1992).

By analogy with Equation (2), lymph formation in pleural and peritoneal lymphatic networks may be quantified as:

(3) $$J_{\text{lymph}}=K_{\text{lymph}}\;\left(P_{\text{lymph}}–P_{\mathrm{liq}}\right)=K_{\text{lymph}}\cdot \Delta P_{\mathrm{TM}}$$

where *P*_liq_ is the pleural or peritoneal fluid pressure. The regional distribution of *P*_liq_ values in different areas of the pleural cavity has been extensively measured in mammals, allowing the distinction between various pleural regions (Miserocchi et al. 1990, 1981, 1982). Conversely, *P*_lymph_ values have been recorded only in intercostal (Moriondo et al. 2005) and diaphragmatic lymphatics (Negrini et al. 2004; Moriondo et al. 2008). Studies performed in spontaneously breathing rodents revealed that lymphatic drainage of pleural fluid and solutes occurs in the costal, diaphragmatic, and mediastinal regions, with the latter providing the largest contribution (Negrini et al. 1985) in terms of percentage of fluid removed relative to the total egress rate. In terms of Δ*P*_TM_, the only contribution to pleural fluid drainage that can be experimentally analyzed is that of the intercostal lymphatics, in which both *P*_lymph_ and *P*_liq_ have been measured during spontaneous breathing (Moriondo et al. 2005; Miserocchi et al. 1982). Although collected from different rodent species, the available data indicate that both costal *P*_liq_ (Miserocchi et al. 1981, 1982) and, to an even greater extent, intercostal *P*_lymph_ (Moriondo et al. 2005) significantly decrease during inspiration, so that a negative Δ*P*_TM_, which drives the entry of pleural fluid into stomata and lacunae, develops throughout the entire spontaneous respiratory cycle. In the intact closed chest, in the supradiaphragmatic region, end‐expiratory *P*_lymph_ and *P*_liq_ values average approximately −9 mmHg and −4 mmHg, respectively (Negrini and Del Fabbro 1999), demonstrating the capability of diaphragmatic lymphatics to absorb pleural fluid. At present, no information is available on inspiratory diaphragmatic *P*_lymph_ changes. However, since during inspiration supradiaphragmatic *P*_liq_ values may drop to −15 mmHg (Miserocchi et al. 1981, 1982), one may reasonably hypothesize that to ensure continuous pleural fluid drainage, a similar or even greater decrease in *P*_lymph_ should occur.

A significant fraction of the total pleural lymphatic outflow occurs in the mediastinal region (Negrini et al. 1985), where the parietal pleura is richly supplied by pericardiac lymphatics and stomata (Wang 1975). In this region, which is directly exposed to powerful myocardial contractions, pleural fluid pressure attains, at both end‐expiratory and at end‐inspiratory lung volumes, the most subatmospheric values measured in the pleural cavity (Miserocchi et al. 1981, 1982), suggesting that high radial stresses and tissue displacements experienced by lymphatic structures are advantageously exploited to sustain efficient lymphatic drainage of the cavity.

In the peritoneal cavity, the mechanisms of fluid filtration and removal closely follow those observed in the pleural space, albeit with important quantitative differences. Serosal peritoneal fluid is filtered down a transmesothelial pressure gradient (Negrini et al. 1990, 1993), favored by peritoneal fluid pressure that, particularly in the suprahepatic region, tends to be subatmospheric, although less negative than in the pleural space (Miserocchi et al. 1982). Despite similar mesothelial hydraulic conductivities and filtering pressure gradients, net fluid filtration (~1.5 mL/kg·h) is approximately 15‐fold higher in the peritoneal than in the pleural space (Negrini et al. 1990, 1993), due to the significantly larger filtering mesothelial surface. Indeed, while in the pleural space filtration occurs only through the parietal pleura (Miserocchi et al. 1988), in the peritoneum the filtering surface extends to all visceral organs, whose mesothelium is supplied by systemic capillaries (Negrini et al. 1990, 1993). Therefore, peritoneal fluid drainage occurs entirely through the lymphatic vasculature, whose draining capability greatly exceeds that of pleural lymphatics to accommodate the much higher filtration rate. The marked difference between the tubular, circuit‐like lacunae observed on the pleural side of the diaphragm and the large, flattened, lacy‐ladder shaped lymphatic structures viewed on the peritoneal side (Figure 2A',B') likely reflects the greater capacity of the peritoneal compared with the pleural diaphragmatic lymphatic reservoirs.

A major functional difference between pleural and peritoneal cavities is their tolerance to increased serosal fluid volume, which is far more tightly regulated in the former. Indeed, even a small expansion of pleural fluid volume (pleural effusion) threatens the tight coupling between the lung and the chest wall, potentially leading to respiratory failure. By contrast, this scenario does not apply to the peritoneal cavity, where serosal fluid primarily serves to ensure lubrication of the abdominal viscera and to facilitate their reciprocal sliding.

### 5.1 Transdiaphragmatic Pleuro‐Peritoneal Connections

**FIGURE 8:**
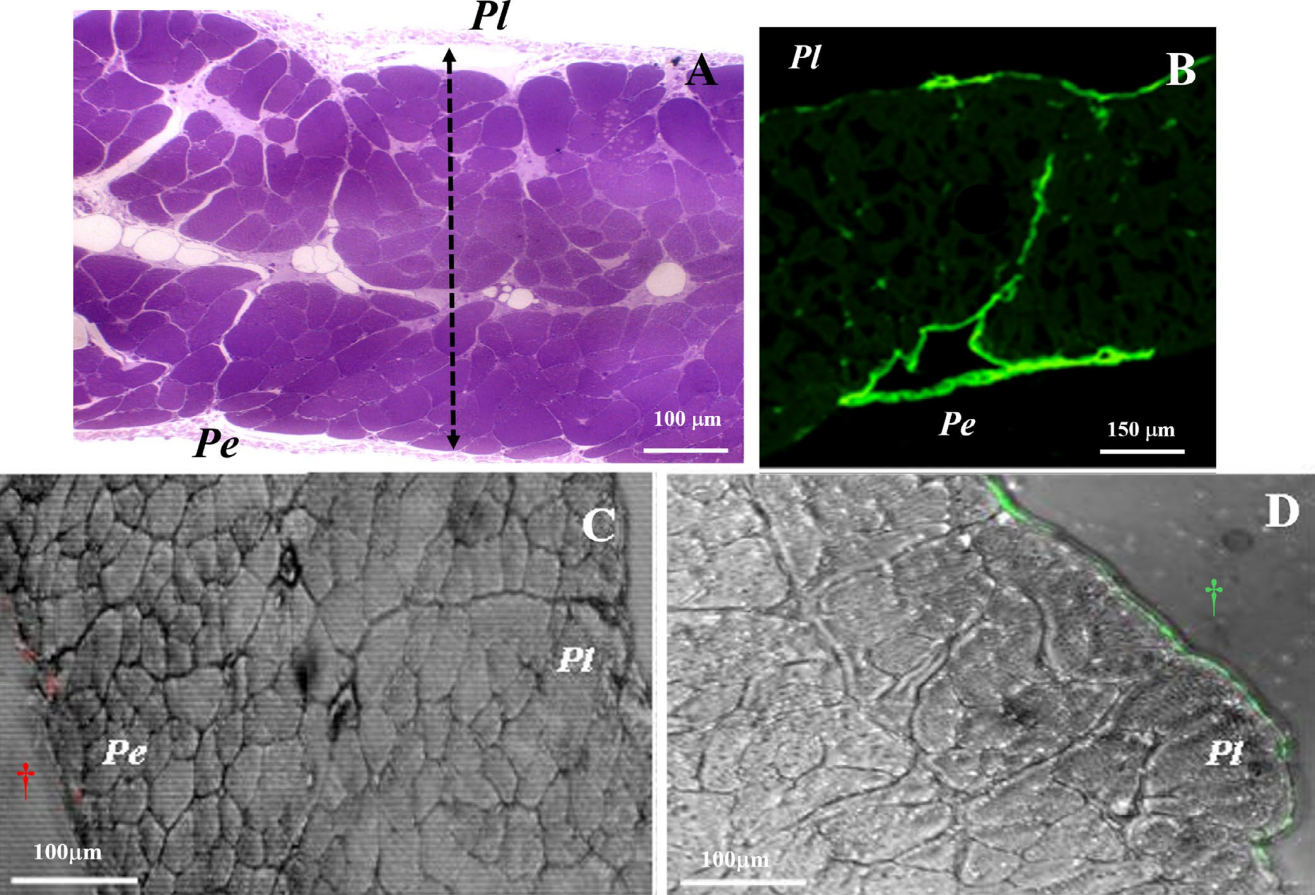
Transperitoneal routes through diaphragmatic lymphatic vasculature. Semithin transverse cross‐section of the entire rat diaphragm (A) and low‐magnification confocal image of anti‐LYVE‐1 fluorescently stained mouse diaphragm (B). These images suggest the existence of potential pleuro‐peritoneal lymphatic connections. However, transverse sections of the rat diaphragm following induction of experimental ascites (C) or pleural effusion (D) by injection of TRITC‐ (red spots) or FITC‐ (green spots) conjugated dextrans into the peritoneal or pleural cavity, respectively, do not provide evidence of net transdiaphragmatic transfer. The dashed line in panel A identifies the pleural‐to‐peritoneal thickness of the diaphragm. Pe, peritoneal side, Pl, pleural side. † injection site.

Although belonging to the same structure and presenting several analogies, the pleural and peritoneal sides of the diaphragm display distinct structural differences (Figure 8). Specifically, the pleural submesothelial interstitium is thicker than the peritoneal one (Grimaldi et al. 2006) and displays a different collagen bundle organization. From the lymphatic system standpoint, the most relevant disparity lies in the markedly different shape and spatial extent of the submesothelial collectors, which are arranged in convoluted tubular ducts and loops on the pleural side, as illustrated by confocal microscopy images in Figure 2. By contrast, on the peritoneal side these structures are organized as broader, flattened, lacy‐ladder shaped reservoirs. In addition, as reported in Figure 8A and clearly demonstrated by anti‐LYVE‐1 fluorescent staining confocal images (Figure 8B), large transverse ducts appear to originate from the superficial lacunae and converge toward the center of the diaphragmatic thickness, thereby establishing a tortuous yet direct connection between the two submesothelial lymphatic structures (Moriondo, Grimaldi, et al. 2007). Accordingly, the key questions are whether: (a) the two cavities are actually functionally connected through this extensive mesh of lymphatic lacunae and collectors; (b) such potential connection allows transmesothelial exchange of fluid and particles; and (c) what is the quantitative contribution of such potential flux to pleural and peritoneal fluid turnover and optimal inter‐organ function under physiological steady‐state or pathophysiological conditions.

Since confocal microscopy analyses, such as the image shown in Figure 8B, reveal the morphology of LYVE‐1 positive structures forming a putative transdiaphragmatic pathway, these issues can be addressed from a functional standpoint. Direct measurements of *P*_liq_ in supradiaphragmatic pleural and subdiaphragmatic peritoneal regions (Negrini et al. 1990; Miserocchi et al. 1981, 1982) have shown that, in the supine position and at any ventrodorsal height, subdiaphragmatic *P*_liq_ is less subatmospheric than supradiaphragmatic *P*_liq_, particularly during inspiration, when pleural *P*_liq_ drops to lower values, while abdominal *P*_liq_ rises as a consequence of the cranio‐caudal diaphragmatic shift (De Troyer et al. 1999; De Troyer and Wilson 2002). Despite the presence of a net peritoneal‐to‐pleural hydraulic gradient, under physiological conditions, serosal fluid does not cross the diaphragm. Indeed, fluid and solutes that enter the lymphatic stomata opening on both sides of the diaphragm are conveyed into deeper lymphatic collectors, without reaching the contralateral serosal side (Moriondo, Grimaldi, et al. 2007). Even in the presence of pleural effusion, which implies an increase in pleural *P*_liq_, fluid absorbed by diaphragmatic lymphatics does not reach peritoneal lymphatic structures (Moriondo, Grimaldi, et al. 2007). This phenomenon may be explained by: (a) diaphragmatic *P*_lymph_ values (Moriondo et al. 2005; Negrini and Del Fabbro 1999; Negrini et al. 2004) that are significantly more subatmospheric than both pleural and peritoneal *P*_liq_, thereby promoting absorption and convergence of fluid from both serosal cavities into deep central lymphatics, from which fluid leaves the diaphragm; and (b) fluid flow conveyed from pleural and peritoneal cavities toward deep lymphatics by unidirectional secondary lymphatic valves (Figure 1B), thereby efficiently minimizing fluid backflow. Taken together, experimental evidence indicates that, despite favorable pressure gradients, transdiaphragmatic percolation of fluid and/or solutes does not occur, at least under physiological conditions. Nevertheless, pathological states, such as infections or inflammation (Lai‐Fook et al. 2005), liver diseases, excessive ascites fluid accumulation, peritoneal dialysis (Nomoto et al. 1989; Chow et al. 2002; Kang and Kim 2009; Kennedy et al. 2011; Chavannes et al. 2014; Alhasan 2019), and/or obesity, conditions often accompanied by the release of tissue proteases, may damage diaphragmatic lymphatic structures, thereby compromising the function of unidirectional valves and/or anchoring filaments that bind lymphatic vessels to the fibrous matrix scaffold, thus causing global impairment of lymphatic function.

## 6 Pulmonary Lymphatics

Given the low hydraulic pressure regime in the pulmonary circulation (Raj and Chen 1986; Negrini, Gonano, and Miserocchi 1992) fluid is continuously filtered into the surrounding interstitium, driven by the highly subatmospheric pulmonary interstitial pressure (Miserocchi et al. 1990, 1991). Under physiological steady‐state conditions, filtration occurs not only across the endothelium of the arteriolar side of the pulmonary capillaries, but also at the venular end, so that fluid egress from the interstitium can occur exclusively through pulmonary lymphatics. Importantly, to ensure adequate gas exchange, the thickness of the alveolo‐capillary membrane must be minimized, a condition achieved by limiting the volume of pericapillary interstitial tissue through: (a) the low hydraulic permeability of the pulmonary endothelial wall (Taylor and Parker 1985), (b) the high interstitial tissue stiffness (Moriondo et al. 2010) and, (c) prompt and efficient lymphatic removal. The limited extent of fluid filtration and the relative “dryness” of the pulmonary parenchyma are evidenced by quantification of pulmonary lymph flow, which amounts to approximately 0.1 mL·min^−1^ 100 g wet tissue weight^−1^ (Albertine et al. 1982), corresponding to only 3% to 15% (Levick and McHale 2003) of total lymph flow in the thoracic duct (approximately 1 mL·h^−1^·Kg^−1^ body weight), with most lymph being formed in the liver (up to ~50%) and in the gastrointestinal tract (up to ~40%).

It has been proposed (Miserocchi et al. 1990, 1991) that pulmonary lymphatics may also be partially involved in pleural fluid drainage. Indeed, a transmesothelial pleural‐to‐pulmonary interstitium pressure gradient exists, driven by the highly subatmospheric pulmonary interstitial pressure. However, absorption of pleural fluid by pulmonary lymphatics is expected to be minimal under physiological conditions, accounting for less than 20% of total pleural fluid egress (Negrini et al. 1985). This reflects the very low hydraulic conductivity of the visceral pleura, due to at least two morphological features: (a) the presence of tight and adherens junctions between mesothelial cells (Wang 1985; Wang et al. 1997), and (b) the thickness and firmness of matrix fibers in its submesothelial interstitium (Albertine et al. 1982). The fluid entering the lung parenchyma through the visceral pleura would ultimately be removed via the complex submesothelial lymphatic network of the visceral pleura, which connects to deeper pulmonary lymphatic collectors (Kuhlman 1997; Finley and Rusch 2011).

The lungs are supplied by an extensive mesh of lymphatics, arranged around almost every airway, artery and vein. Small initial lymphatics, which are devoid of LMCs (Kretschmer et al. 2013; Reed et al. 2019), completely surround bronchioles, alveoli, alveolar ducts, and interlobular septa (Leak and Jamuar 1983; Lee et al. 2021; Trapnell 1970; Schraufnagel 2013). Peribronchial, periarterial, perivenous, saccular, and interalveolar lymphatics drain into bronchial, arterial, and venous lymphatics, which eventually merge into lymphatic collectors equipped with valves and LMCs. From there, lymph reaches the hilum, the caudal mediastinal node (Lee et al. 2021; Hainis et al. 1994), and ultimately empties into the right lymphatic duct. Lymphatic vessels also lie in the thick interstitial layer supporting the visceral pleura (Albertine et al. 1982; Hainis et al. 1994). Unlike those running underneath the parietal mesothelium, which are characterized by stomata directly opening onto the pleural space (Figure 2A), these lymphatics lack stomata and do not directly drain from the pleural cavity.

Lymphatic drainage in the lungs is critically important. Indeed, despite normal alveolar surfactant synthesis, knockout mice lacking the key lymphangiogenic factor VEGFC (Vascular Endothelial Growth Factor C) die at birth, from massive pulmonary edema and respiratory failure (Jakus et al. 2014). Heterozygous mice are also affected and, although to a lesser extent, develop lymphatic hypoplasia and primary lymphedema (Karkkainen et al. 2004). Similar severe impairments are observed in transplanted lungs, in which lymphatic trunks have been surgically resected without the possibility of establishing new patent anastomoses and are therefore prone to developing chronic edema. Accordingly, therapeutic lymphangiogenesis has then been proposed as a post‐surgery approach to promote the growth of new lymphatic vasculature, thereby enhancing clearance of inflammatory cells and hyaluronic acid fragments (Cui et al. 2015). Notably, spontaneous development of newly formed perianastomotic lymphatic meshes has been documented in the spermatic cord, the inguinocrural, inguinoscrotal, inguinotesticular, and brachial regions, in patients undergoing microsurgical canalization of lymphatic collectors aimed at the surgical treatment of severe secondary edema (Mukenge et al. 2007, 2013, 2023). Therapeutic lymphangiogenesis may further enhance such spontaneous reconstructive processes, thus representing a promising therapeutic strategy for chronic lymphedema in transplanted lungs.

Lymphatic impairment and, consequently, progressive development of edema, often accompanied by pleural effusion, can also be observed during mechanical ventilation at positive alveolar pressure. This condition is associated with increased transpulmonary pressure, regional alveolar overdistension, and the sequential activation of tissue metalloproteases (Negrini et al. 2006; Moriondo, Pelosi, et al. 2007). All together, these factors exert a detrimental effect on the three‐dimensional organization of the fibrous interstitial matrix. In particular, the release and/or activation of tissue metalloproteases induces a progressive fragmentation of both structural proteoglycans and those lining the endothelial layers, such as perlecan or laminin (Pelosi and Negrini 2008; Negrini et al. 2008; Moriondo et al. 2012; Marcozzi et al. 2015). Accordingly, mechanical ventilation, by causing potential structural injury and fragmentation of the tissue matrix scaffold, including anchoring filaments, is likely to compromise pulmonary lymphatic performance, thereby exacerbating the development of lung edema.

Pulmonary lymphatics, in particular those supplying the peripheral lung lobules, might represent, by virtue of their structure and size, a potential dissemination route for pollutant macromolecules and amphibolites, or asbestos fibers, from the lung parenchyma, the primary site of infiltration of amphibolites from the alveolar space, to the pulmonary circulation and eventually to the mesothelial layer (Miserocchi et al. 2008). In the latter, amphibole fibers are known to induce processes ranging from chronic inflammation and stomata obstruction (Boutin et al. 1996), to pleural fibrosis, effusion, and ultimately malignant mesothelioma.

## 7 Myocardial Lymphatics

The heart and mediastinum possess an anatomically distinct lymphatic drainage, which appears to be independent of the functionally interconnected intercostal‐diaphragm–lung–pleural‐peritoneal lymphatic complex. The ventricles of the heart are supplied by an extensive network of lymphatic vessels, distributed within the epicardial, subepicardial, and endocardial tissue layers, whereas their presence is relatively sparse among myocardial fibers (Tatin et al. 2017). Both atria show limited evidence of lymphatic vessels (Ware et al. 2026), which are more developed in the left atrium than in the right. Epicardial, myocardial, and subendocardial regions are supplied by an extensive plexus of lymphatic capillaries, embedded within the connective tissue surrounding muscle bundles, which drain fluid toward the subepicardium via intramyocardial vessels. These lymphatic microvessels are essentially endothelialized tissue channels and are apparently not surrounded by LMCs. Myocardial initial lymphatics drain into subepicardial collectors equipped with intraluminal unidirectional valves (Ware et al. 2026; Laine and Allen 1991; Klaourakis et al. 2021), forming the right and left coronary lymphatics, which eventually merge into the principal cardiac lymphatic duct. The latter conveys lymph toward the cardiac lymph node and ultimately into the right lymphatic duct.

The mechanisms sustaining cardiac lymph flow display many features in common with those observed in diaphragmatic lymphatics. In particular, lymph formation and propulsion critically depend on myocardial contraction, with the systole‐to‐diastole duration ratio playing a crucial role (Bradham et al. 1973). As in diaphragmatic initial lymphatics, cardiac lymphatic endothelial cells are connected to extracellular macromolecules, such as fibronectin and Type 1 collagen, through anchoring filaments composed of emilin‐1 and fibrillin (Mehlhorn et al. 2001). Accordingly, as in all other thoracic tissues, these molecular structures enable myocardial lymphatic vessels to exploit phasic tissue displacements and mechanical stresses generated during the cardiac contraction cycle to sustain lymph flow and adapt to local fluid drainage requirements (Mehlhorn et al. 2001). To date, experimental challenges have limited detailed hydrodynamic investigations of cardiac lymphatic function under physiological steady‐state conditions. Nevertheless, extensive clinical and experimental evidences indicate that malfunction or insufficiency of cardiac lymphatics play a significant role in the development or exacerbation of several myocardial pathophysiological conditions, including myocardial edema (Mehlhorn et al. 2001; Nilsson et al. 2001; Friedrich 2010), ischemia (Nilsson et al. 2001), myocarditis (Baeßler et al. 2015), nonischemic cardiomyopathy (Nishii et al. 2014), acute myocardial fibrillation (Verbrugge et al. 2017), cardiac amyloidosis (Kotecha et al. 2018), and Takotsubo cardiomyopathy (Abdel‐Aty et al. 2009). Lymphatic insufficiency reduces interstitial fluid clearance, leading to the development of local interstitial edema, thereby indirectly impairing cardiac contractility (Ludwig et al. 1997) and increasing ventricular stiffness (Laine and Allen 1991; Cross et al. 1961; Desai et al. 2008). The latter effect has also been observed following experimental blockade of lymphatic outflow, a condition that promotes interstitial fibrosis and severe diastolic dysfunction (Laine and Allen 1991; Desai et al. 2008). Further support for the pathological relevance of myocardial lymphatic impairment comes from studies showing that experimentally induced lymphangiogenesis improves the clinical outcome in several models of myocardial disease (Klotz et al. 2015; Henri et al. 2016; Song et al. 2020; Heron et al. 2023). Similarly, as observed after microsurgical canalization of previously damaged lymphatic collectors (Mukenge et al. 2007, 2013, 2023), recovery following myocardial ischemia is characterized by both tissue remodeling and local lymphangiogenesis within the infarcted region (Henri et al. 2016; van Amerongen et al. 2007; Nahrendorf et al. 2007; Frantz et al. 2013; Vieira et al. 2018).

## 8 Flow Modulation and Saturation in Thoracic Lymphatics

**FIGURE 9:**
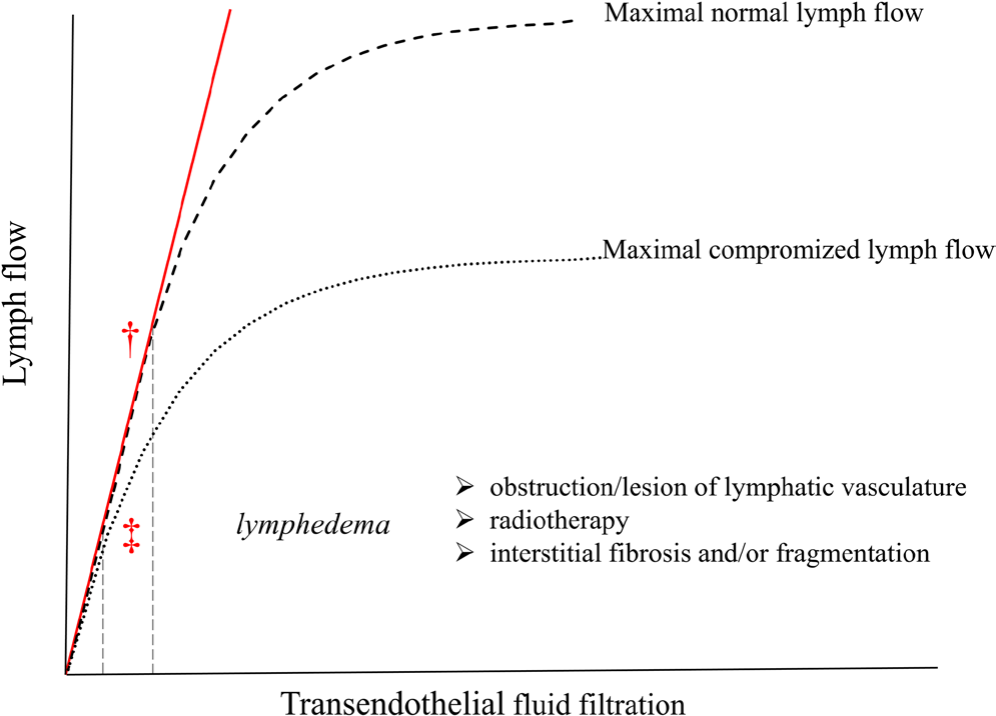
Lymph flow modulation. Graph illustrating the dependence of lymph flow on transendothelial or transmesothelial fluid filtration. The origin of axes identifies the steady‐state condition, in which lymph flow exactly matches filtration rate and tissue fluid volume remains constant. If transmembrane filtration increases, lymph flow (dashed line) automatically rises at a similar rate along the solid red line, thereby maintaining steady‐state interstitial fluid volume. Although lymph drainage may increase several‐fold, even under physiological conditions, it progressively reaches a maximal value, defined by the specific capacitance of the lymphatic vasculature and by the efficiency of the propelling mechanisms. Interstitial fluid accumulation begins when the filtration rate exceeds lymphatic drainage capacity, a condition identified in the graph by divergence of the solid red line from the black dashed line (red †). Damage to or impairment of the lymphatic vasculature (dotted line) markedly reduces maximal lymph flow, leading to the development of lymphedema even at relatively low filtration rates, as illustrated by the earlier divergence of the dotted line from the red solid one (red ‡).

It has long been stated that the lymphatic vasculature represents a sort of “emergency route” for the egress of fluid, particles, and cells, aimed at counteracting significant imbalance in tissue fluid and solute content. However, this view underestimates the pivotal role played by lymphatics. Indeed, lymphatics recruitment does not occur only after the attainment of a hazardous tissue volume threshold, but rather operates continuously, on a “drop‐by‐drop” basis, thereby preventing excessive tissue swelling from persisting for a prolonged period. Over the years, several evidence from studies of the thoracic duct (Brace and Power 1981), lungs (Drake et al. 1981), mesentery (Benoit et al. 1989), bat wing (Hogan and Unthank 1986), and rat tail (Aarli et al. 1991), have demonstrated the ability of the lymphatic vasculature to adapt local lymph flow to the drainage requirements of a given tissue. According to Equation (2), lymphatic flow from a tissue depends on the tissue‐to‐lymphatic lumen pressure gradient and on lymphatic conductance: an increase in either parameter results in an increase in lymph outflow (Figure 9), which counteracts the fluid perturbation, and thereby prevents fluid accumulation. The Δ*P*_TM_ reflects the combined effects of intrinsic and extrinsic forces acting on the vessel lumen, whereas lymphatic conductance *K*_lymph_ depends on the density and the degree of expansion of the initial lymphatics' inlets, as modulated by stretching of the anchoring filaments. For a given *K*_lymph_, net lymphatic egress rate is directly proportional to Δ*P*_TM_, which in turn increases automatically when interstitial pressure *P*_int_ rises. This behavior was first described by Arthur Guyton's original studies (Guyton et al. 1971) and has been validated in the pleural space, where any increase in *P*_liq_ associated with pleural effusion is promptly compensated by an increased lymphatic outflow (Miserocchi et al. 1993; D. Negrini 2006). The passive response of the lymphatic vasculature to increased tissue fluid content is tightly connected to the mechanical properties of the tissue itself. Indeed, for any given increment in tissue fluid volume, *P*_int_ increases more markedly in tissues characterized by a dense and stiff three‐dimensional scaffold, such as the pulmonary parenchyma (Miserocchi et al. 1990, 1991), than in tissues with looser matrix organization, such as the mesentery. In the lung, even small increments in tissue fluid volume, as occur during the development of mild interstitial edema, may cause a significant increase in local *P*_int_, reaching values of up to + 11 mmHg (Miserocchi et al. 1990, 1991), thereby triggering a lymphatic response that immediately and efficiently offsets the local fluid perturbation.

In thoracic tissues, in addition to these automatic fluid‐dynamic adjustments and to the mechanisms underlying lymphatic pacemaking (Bridenbaugh et al. 2003; Davis et al. 2024; Maisel and Outtz Reed 2025), lymphatic function can be further modulated by local factors, such as interstitial tissue osmolarity (Solari et al. 2018, 2023; Solari, Marcozzi, Negrini, and Moriondo 2020), local tissue temperature (Solari et al. 2017; Solari, Marcozzi, Bistoletti, et al. 2020) and the interstitial concentration of low‐density lipoproteins (Solari, Marcozzi, Bartolini, et al. 2020). Indeed, spontaneously contracting lymphatic collectors supplying diaphragmatic tissue exposed to a hyperosmolar microenvironment exhibit a reduction in spontaneous contractile activity, with a depressed contraction rate and flow. Conversely, collectors exposed to a hyposmolar environment display a rapid and transient increase in contraction frequency, followed by a long‐term reduction.

Tissue temperature also plays a critical role in lymph flow modulation, as demonstrated by comparative analyses between thoracic and dermal lymphatics, which show maximal contractile frequency, and therefore maximal flow, at their respective physiological environmental temperature, which is lower for distal dermal lymphatics than for diaphragmatic ones located within the thermal core (Solari, Marcozzi, Bistoletti, et al. 2020). Notably, experimental data indicate that dermal lymphatics cannot operate efficiently at temperature ranges that are optimal for diaphragmatic lymphatics, and vice versa. Osmolarity‐ and temperature‐dependent adjustments of intrinsic lymphatic contractility have been shown to involve ion channels, such as TRPV1 and TRPV4 (transient receptor potential cation channels, subfamily V, members 1 and 4) and VRACs (volume‐regulated anion channels) (Eggermont et al. 2001). TRPV channels mediate responses to mild local temperature variations, cell swelling or shrinkage, and shear stress (Moriondo et al. 2015; Strotmann et al. 2000; Liedtke et al. 2000; Wu et al. 2007), whereas VRACs are activated by the hyposmotic microenvironment (Eggermont et al. 2001).

Lymphatic flow may increase up to 10–20 fold (Miserocchi et al. 1993) relative to the baseline value, until attaining its maximum level, often referred to as lymphatic saturation. Importantly, although lymph removal is maximal at saturation, it becomes insufficient to meet increased tissue demands; consequently, uncontrolled edema may progress while *P*_int_ shifts toward more positive values. At this stage, in most tissues, including the lung, further increases in *P*_int_ limit additional interstitial swelling primarily by driving excess fluid into the venous end of blood capillaries. The maximal absorptive capability of a lymphatic district is limited by functional saturation or structural damages (Miserocchi et al. 1993; Miserocchi 2009), so that lymphatic outflow can no longer compensate for excessive fluid inflow into the tissue (Figure 9). This condition has been documented in several tissues and in serosal cavities, both in experimental animals and human subjects (Browse et al. 2003). Secondary lymphedema represents a paradigmatic example of this phenomenon, as its development typically follows obstruction or removal of lymphatic segments, or lymphatic damage and inefficiency after trauma, invasive surgery, and/or radiation therapy (Browse et al. 2003). Some of these severely debilitating conditions can be effectively treated by recanalizing disrupted lymphatic pathways through lympho‐venous or lympho‐lymphatic anastomoses, an approach successfully employed in the microsurgical treatment of advanced secondary lymphedema of the upper and lower limbs and external genitalia (Mukenge et al. 2007, 2013, 2023).

## 9 Conclusion

Analysis of the performance of the lymphatic vasculature in thoracic tissues highlights how this component of the cardiovascular system, often undervalued or even neglected, is essential not only for maintaining fluid and solute homeostasis, but also for supporting the overall physiological function of thoracic organs. Indeed, the lymphatic vasculature does not behave as a set of purely passive conduits; rather, it exploits a diverse and integrated repertoire of interorgan strategies to achieve and preserve appropriate fluid and solute balance. In thoracic tissues, local morphology, three‐dimensional organization, fluid dynamics, and the mechanisms driving lymph propulsion through the circuit are not uniform. Instead of being uniform, these features are finely adapted to exploit regional tissue anatomy, mechanical properties, and hydraulic demands. Notably, and to a greater extent than in other body districts, the relevance of the lymphatic vasculature in thoracic tissues is not limited to counteracting tissue fluid imbalances. Rather, it extends to functions critically dependent on tissue hydration, ranging from maintaining the proper lung–chest wall coupling to supporting efficient pulmonary gas exchange. Taken together, the lymphatic vasculature of thoracic tissues provides a compelling example of how apparently independent body functions can cooperate synergistically to ensure optimal organ‐specific and interorgan performance.

## Funding

This work was supported by the Applied Research Platform of the Center for Research and Technological Transfer (CRIETT) of the University of Insubria.

## Disclosure

Permission to reproduce material from other sources: The Figures have been either modified with respect to the already published originals or have never been published elsewhere.

## Ethics Statement

The authors have nothing to report.

## Consent

The authors have nothing to report.

## Conflicts of Interest

The authors declare no conflicts of interest.

## Data Availability

Data sharing not applicable to this article as no datasets were generated or analysed during the current study.

## References

[cph470143-bib-0001] Aarli, V. , R. K. Reed , and K. Aukland . 1991. “Effect of Longstanding Venous Stasis and Hypoproteinaemia on Lymph Flow in the Rat Tail.” Acta Physiologica Scandinavica 142: 1–9. 10.1111/j.1748-1716.1991.tb09122.x. 1877357

[cph470143-bib-0002] Abdel‐Aty, H. , M. Cocker , and M. G. Friedrich . 2009. “Myocardial Edema Is a Feature of Tako‐Tsubo Cardiomyopathy and Is Related to the Severity of Systolic Dysfunction: Insights From T2‐Weighted Cardiovascular Magnetic Resonance.” International Journal of Cardiology 132: 291–293. 10.1016/j.ijcard.2007.08.102. 18086501

[cph470143-bib-0003] Adamson, R. H. , and C. C. Michel . 1993. “Pathways Through the Intercellular Clefts of Frog Mesenteric Capillaries.” Journal of Physiology 466: 303–327. 8410696 PMC1175480

[cph470143-bib-0004] Albertine, K. H. , J. P. Wiener‐Kronish , P. J. Roos , and N. C. Staub . 1982. “Structure, Blood Supply, and Lymphatic Vessels of the Sheep's Visceral Pleura.” American Journal of Anatomy 165: 277–294. 10.1002/aja.1001650305. 7180815

[cph470143-bib-0005] Alhasan, K. A. 2019. “Recurrent Hydrothorax in a Child on Peritoneal Dialysis: A Case Report and Review of the Literature.” Clinical Case Reports 7: 149–151. 10.1002/ccr3.1936. 30656030 PMC6333082

[cph470143-bib-0006] Allen, J. M. , N. G. McHale , and B. M. Rooney . 1983. “Effect of Norepinephrine on Contractility of Isolated Mesenteric Lymphatics.” American Journal of Physiology 244: H479–H486. 10.1152/ajpheart.1983.244.4.H479. 6837752

[cph470143-bib-0007] Aspelund, A. , S. Antila , S. T. Proulx , et al. 2015. “A Dural Lymphatic Vascular System That Drains Brain Interstitial Fluid and Macromolecules.” Journal of Experimental Medicine 212: 991–999. 10.1084/jem.20142290. 26077718 PMC4493418

[cph470143-bib-0008] Aukland, K. , and R. K. Reed . 1993. “Interstitial‐Lymphatic Mechanisms in the Control of Extracellular Fluid Volume.” Physiological Reviews 73: 1–78. 10.1152/physrev.1993.73.1.1. 8419962

[cph470143-bib-0009] Baeßler, B. , F. Schaarschmidt , A. Dick , et al. 2015. “Mapping Tissue Inhomogeneity in Acute Myocarditis: A Novel Analytical Approach to Quantitative Myocardial Edema Imaging by T2‐Mapping.” Journal of Cardiovascular Magnetic Resonance 17: 115. 10.1186/s12968-015-0217-y. 26700020 PMC4690253

[cph470143-bib-0010] Benoit, J. N. , D. C. Zawieja , A. H. Goodman , and H. J. Granger . 1989. “Characterization of Intact Mesenteric Lymphatic Pump and Its Responsiveness to Acute Edemagenic Stress.” American Journal of Physiology 257: H2059–H2069. 10.1152/ajpheart.1989.257.6.H2059. 2603989

[cph470143-bib-0011] Bertram, C. D. , and M. J. Davis . 2023. “An Enhanced 3D Model of Intravascular Lymphatic Valves to Assess Leaflet Apposition and Transvalvular Differences in Wall Distensibility.” Biology 12: 379. 10.3390/biology12030379. 36979071 PMC10044971

[cph470143-bib-0012] Biel, M. , C. Wahl‐Schott , S. Michalakis , and X. Zong . 2009. “Hyperpolarization‐Activated Cation Channels: From Genes to Function.” Physiological Reviews 89: 847–885. 10.1152/physrev.00029.2008. 19584315

[cph470143-bib-0013] Boutin, C. , P. Dumortier , F. Rey , J. R. Viallat , and P. De Vuyst . 1996. “Black Spots Concentrate Oncogenic Asbestos Fibers in the Parietal Pleura. Thoracoscopic and Mineralogic Study.” American Journal of Respiratory and Critical Care Medicine 153: 444–449. 10.1164/ajrccm.153.1.8542156. 8542156

[cph470143-bib-0014] Brace, R. A. , and G. G. Power . 1981. “Thoracic Duct Lymph Flow and Protein Flux Dynamics: Responses to Intravascular Saline.” American Journal of Physiology 240: R282–R288. 10.1152/ajpregu.1981.240.5.R282. 7235046

[cph470143-bib-0015] Bradham, R. R. , E. F. Parker , and W. B. Greene . 1973. “Lymphatics of the Atrioventricular Valves.” Archives of Surgery 106: 210–213. 10.1001/archsurg.1973.01350140068019. 4686523

[cph470143-bib-0016] Bridenbaugh, E. A. , A. A. Gashev , and D. C. Zawieja . 2003. “Lymphatic Muscle: A Review of Contractile Function.” Lymphatic Research and Biology 1: 147–158. 10.1089/153968503321642633. 15624422

[cph470143-bib-0017] Browse, N. , K. G. Burnand , and P. S. Mortimer . 2003. Diseases of the Lymphatics. Arnold.

[cph470143-bib-0018] Castenholz, A. 1986. “Corrosion Cast Technique Applied in Lymphatic Pathways.” Scanning Electron Microscopy 2, no. 2: 599–605. 3797998

[cph470143-bib-0019] Castenholz, A. 1989. “Interpretation of Structural Patterns Appearing on Corrosion Casts of Small Blood and Initial Lymphatic Vessels.” Scanning Microscopy 3: 315–325. 2740869

[cph470143-bib-0020] Cha, E. M. , and P. Sirijintakarn . 1976. “Anatomic Variation of the Thoracic Duct and Visualization of Mediastinal Lymph Nodes: A Lymphographic Study.” Radiology 119: 45–48. 10.1148/119.1.45. 1257453

[cph470143-bib-0021] Chavannes, M. , A. P. Sharma , R. N. Singh , R. H. Reid , and G. Filler . 2014. “Diagnosis by Peritoneal Scintigraphy of Peritoneal Dialysis‐Associated Hydrothorax in an Infant.” Peritoneal Dialysis International 34: 140–143. 10.3747/pdi.2012.00077. 24525610 PMC3923712

[cph470143-bib-0022] Chen, M. , M. P. Marinkovich , A. Veis , et al. 1997. “Interactions of the Amino‐Terminal Noncollagenous (NC1) Domain of Type VII Collagen With Extracellular Matrix Components. A Potential Role in Epidermal‐Dermal Adherence in Human Skin.” Journal of Biological Chemistry 272: 14516–14522. 10.1074/jbc.272.23.14516. 9169408

[cph470143-bib-0023] Chow, K. M. , C. C. Szeto , T. Y.‐H. Wong , and P. K.‐T. Li . 2002. “Hydrothorax Complicating Peritoneal Dialysis: Diagnostic Value of Glucose Concentration in Pleural Fluid Aspirate.” Peritoneal Dialysis International 22: 525–528. 12322829

[cph470143-bib-0024] Contarino, C. , and E. F. Toro . 2018. “A One‐Dimensional Mathematical Model of Collecting Lymphatics Coupled With an Electro‐Fluid‐Mechanical Contraction Model and Valve Dynamics.” Biomechanics and Modeling in Mechanobiology 17: 1687–1714. 10.1007/s10237-018-1050-7. 30006745

[cph470143-bib-0025] Cross, C. E. , P. A. Rieben , and P. F. Salisbury . 1961. “Influence of Coronary Perfusion and Myocardial Edema on Pressure‐Volume Diagram of Left Ventricle.” American Journal of Physiology 201: 102–108. 10.1152/ajplegacy.1961.201.1.102. 13696613

[cph470143-bib-0026] Cui, Y. , K. Liu , M. E. Monzon‐Medina , et al. 2015. “Therapeutic Lymphangiogenesis Ameliorates Established Acute Lung Allograft Rejection.” Journal of Clinical Investigation 125: 4255–4268. 10.1172/JCI79693. 26485284 PMC4639995

[cph470143-bib-0027] Curry, F. 1984. “Mechanism and Thermodynamics of Transcapillary Exchange.” In Handbook of Physiology. The Cardiovascular System. Microcirculation, 309–374. American Physiological Society.

[cph470143-bib-0029] Davis, M. J. , J. A. Castorena‐Gonzalez , M. Li , et al. 2024. “Connexin‐45 Is Expressed in Mouse Lymphatic Endothelium and Required for Lymphatic Valve Function.” JCI Insight 9: e169931. 10.1172/jci.insight.169931. 39074069 PMC11343601

[cph470143-bib-0030] Davis, M. J. , E. Rahbar , A. A. Gashev , D. C. Zawieja , and J. E. J. Moore . 2011. “Determinants of Valve Gating in Collecting Lymphatic Vessels From Rat Mesentery.” American Journal of Physiology. Heart and Circulatory Physiology 301: H48–H60. 10.1152/ajpheart.00133.2011. 21460194 PMC3129915

[cph470143-bib-0028] Davis, M. J. , and S. D. Zawieja . 2024. “Pacemaking in the Lymphatic System.” Journal of Physiology. 10.1113/JP284752. PMC13235532 38520402

[cph470143-bib-0031] De Troyer, A. , A. Legrand , and T. A. Wilson . 1999. “Respiratory Mechanical Advantage of the Canine External and Internal Intercostal Muscles.” Journal of Physiology 518: 283–289. 10.1111/j.1469-7793.1999.0283r.x. 10373709 PMC2269416

[cph470143-bib-0032] De Troyer, A. , and T. A. Wilson . 2002. “Coupling Between the Ribs and the Lung in Dogs.” Journal of Physiology 540: 231–236. 10.1113/jphysiol.2001.013319. 11927682 PMC2290201

[cph470143-bib-0033] Desai, K. V. , G. A. Laine , R. H. Stewart , et al. 2008. “Mechanics of the Left Ventricular Myocardial Interstitium: Effects of Acute and Chronic Myocardial Edema.” American Journal of Physiology. Heart and Circulatory Physiology 294: H2428–H2434. 10.1152/ajpheart.00860.2007. 18375722

[cph470143-bib-0034] DiFrancesco, D. 1981. “A New Interpretation of the Pace‐Maker Current in Calf Purkinje Fibres.” Journal of Physiology 314: 359–376. 10.1113/jphysiol.1981.sp013713. 6273533 PMC1249439

[cph470143-bib-0035] DiFrancesco, D. 1982. “Block and Activation of the Pace‐Maker Channel in Calf Purkinje Fibres: Effects of Potassium, Caesium and Rubidium.” Journal of Physiology 329: 485–507. 10.1113/jphysiol.1982.sp014315. 6292407 PMC1224792

[cph470143-bib-0036] DiFrancesco, D. , F. Porciatti , D. Janigro , et al. 1991. “Block of the Cardiac Pacemaker Current (If) in the Rabbit Sino‐Atrial Node and in Canine Purkinje Fibres by 9‐Amino‐1,2,3,4‐Tetrahydroacridine.” Pflügers Archiv 417: 611–615. 10.1007/BF00372959. 2057325

[cph470143-bib-0038] Drake, R. , T. Adair , D. Traber , and J. Gabel . 1981. “Contamination of Caudal Mediastinal Node Efferent Lymph in Sheep.” American Journal of Physiology 241: H354–H357. 10.1152/ajpheart.1981.241.3.H354. 7282944

[cph470143-bib-0039] Eggermont, J. , D. Trouet , I. Carton , and B. Nilius . 2001. “Cellular Function and Control of Volume‐Regulated Anion Channels.” Cell Biochemistry and Biophysics 35: 263–274. 10.1385/CBB:35:3:263. 11894846

[cph470143-bib-0040] Finley, D. J. , and V. W. Rusch . 2011. “Anatomy of the Pleura.” Thoracic Surgery Clinics 21: 157–163. 10.1016/j.thorsurg.2010.12.001. 21477764

[cph470143-bib-0041] Frantz, S. , U. Hofmann , D. Fraccarollo , et al. 2013. “Monocytes/Macrophages Prevent Healing Defects and Left Ventricular Thrombus Formation After Myocardial Infarction.” FASEB Journal 27: 871–881. 10.1096/fj.12-214049. 23159933

[cph470143-bib-0042] Friedrich, M. G. 2010. “Myocardial Edema–A New Clinical Entity?” Nature Reviews. Cardiology 7: 292–296. 10.1038/nrcardio.2010.28. 20309007

[cph470143-bib-0043] Granger, H. J. , G. Laine , G. Barnes , and R. Lewis . 1984. “Dynamics and Control of Transmicrovascular Fluid Exchange.” In Edema, 189–228. Raven Press.

[cph470143-bib-0044] Grimaldi, A. , A. Moriondo , L. Sciacca , M. L. Guidali , G. Tettamanti , and D. Negrini . 2006. “Functional Arrangement of Rat Diaphragmatic Initial Lymphatic Network.” American Journal of Physiology. Heart and Circulatory Physiology 291: H876–H885. 10.1152/ajpheart.01276.2005. 16489104

[cph470143-bib-0045] Guyton, A. 1963. “A Concept of Negative Interstitial Pressure Based on Pressure in Implanted Perforated Capsules.” Circulation Research 12: 399–414. 13951514 10.1161/01.res.12.4.399

[cph470143-bib-0046] Guyton, A. C. , H. J. Granger , and A. E. Taylor . 1971. “Interstitial Fluid Pressure.” Physiological Reviews 51: 527–563. 10.1152/physrev.1971.51.3.527. 4950077

[cph470143-bib-0047] Guyton, A. C. , A. E. Taylor , and H. J. Granger . 1975. “Dynamics and Control of Body Fluids.” In Circulatory Physiology II. Saunders W.B.

[cph470143-bib-0048] Hainis, K. D. , J. I. Sznajder , and D. E. Schraufnagel . 1994. “Lung Lymphatics Cast From the Airspace.” American Journal of Physiology 267: L199–L205. 10.1152/ajplung.1994.267.2.L199. 8074244

[cph470143-bib-0049] Hald, B. O. , J. A. Castorena‐Gonzalez , S. D. Zawieja , P. Gui , and M. J. Davis . 2018. “Electrical Communication in Lymphangions.” Biophysical Journal 115: 936–949. 10.1016/j.bpj.2018.07.033. 30143234 PMC6127464

[cph470143-bib-0050] Henri, O. , C. Pouehe , M. Houssari , et al. 2016. “Selective Stimulation of Cardiac Lymphangiogenesis Reduces Myocardial Edema and Fibrosis Leading to Improved Cardiac Function Following Myocardial Infarction.” Circulation 133: 1484–1497. 10.1161/CIRCULATIONAHA.115.020143. 26933083

[cph470143-bib-0051] Heron, C. , A. Dumesnil , M. Houssari , et al. 2023. “Regulation and Impact of Cardiac Lymphangiogenesis in Pressure‐Overload‐Induced Heart Failure.” Cardiovascular Research 119: 492–505. 10.1093/cvr/cvac086. 35689481 PMC10064842

[cph470143-bib-0052] Hogan, R. D. , and J. L. Unthank . 1986. “The Initial Lymphatics as Sensors of Interstitial Fluid Volume.” Microvascular Research 31: 317–324. 10.1016/0026-2862(86)90020-8. 3713549

[cph470143-bib-0053] Iliff, J. J. , M. Wang , Y. Liao , et al. 2012. “A Paravascular Pathway Facilitates CSF Flow Through the Brain Parenchyma and the Clearance of Interstitial Solutes, Including Amyloid β.” Science Translational Medicine 4: 147ra111. 10.1126/scitranslmed.3003748. PMC3551275 22896675

[cph470143-bib-0054] Jakus, Z. , J. P. Gleghorn , D. R. Enis , et al. 2014. “Lymphatic Function Is Required Prenatally for Lung Inflation at Birth.” Journal of Experimental Medicine 211: 815–826. 10.1084/jem.20132308. 24733830 PMC4010903

[cph470143-bib-0055] Jessen, N. A. , A. S. F. Munk , I. Lundgaard , and M. Nedergaard . 2015. “The Glymphatic System: A Beginner's Guide.” Neurochemical Research 40: 2583–2599. 10.1007/s11064-015-1581-6. 25947369 PMC4636982

[cph470143-bib-0056] Kang, T. W. , and C. K. Kim . 2009. “Pleuroperitoneal Communication of Peritoneal Dialysis Demonstrated by Multidetector‐Row CT Peritoneography.” Abdominal Imaging 34: 780–782. 10.1007/s00261-008-9468-5. 18972150

[cph470143-bib-0057] Karkkainen, M. J. , P. Haiko , K. Sainio , et al. 2004. “Vascular Endothelial Growth Factor C Is Required for Sprouting of the First Lymphatic Vessels From Embryonic Veins.” Nature Immunology 5: 74–80. 10.1038/ni1013. 14634646

[cph470143-bib-0058] Kennedy, C. , C. McCarthy , S. Alken , et al. 2011. “Pleuroperitoneal Leak Complicating Peritoneal Dialysis: A Case Series.” International Journal of Nephrology 2011: 526753. 10.4061/2011/526753. 21876802 PMC3161202

[cph470143-bib-0059] Kirkpatrick, C. T. , and N. G. McHale . 1977. “Electrical and Mechanical Activity of Isolated Lymphatic Vessels [Proceedings].” Journal of Physiology 272: 33P–34P. 592136

[cph470143-bib-0060] Klaourakis, K. , J. M. Vieira , and P. R. Riley . 2021. “The Evolving Cardiac Lymphatic Vasculature in Development, Repair and Regeneration.” Nature Reviews. Cardiology 18: 368–379. 10.1038/s41569-020-00489-x. 33462421 PMC7812989

[cph470143-bib-0061] Klotz, L. , S. Norman , J. M. Vieira , et al. 2015. “Cardiac Lymphatics Are Heterogeneous in Origin and Respond to Injury.” Nature 522: 62–67. 10.1038/nature14483. 25992544 PMC4458138

[cph470143-bib-0062] Koh, L. , A. Zakharov , and M. Johnston . 2005. “Integration of the Subarachnoid Space and Lymphatics: Is It Time to Embrace a New Concept of Cerebrospinal Fluid Absorption?” Cerebrospinal Fluid Research 2: 6. 10.1186/1743-8454-2-6. 16174293 PMC1266390

[cph470143-bib-0063] Kotecha, T. , A. Martinez‐Naharro , T. A. Treibel , et al. 2018. “Myocardial Edema and Prognosis in Amyloidosis.” Journal of the American College of Cardiology 71: 2919–2931. 10.1016/j.jacc.2018.03.536. 29929616

[cph470143-bib-0064] Kretschmer, S. , I. Dethlefsen , S. Hagner‐Benes , L. M. Marsh , H. Garn , and P. König . 2013. “Visualization of Intrapulmonary Lymph Vessels in Healthy and Inflamed Murine Lung Using CD90/Thy‐1 as a Marker.” PLoS One 8: e55201. 10.1371/journal.pone.0055201. 23408960 PMC3568125

[cph470143-bib-0065] Kuhlman, J. E. 1997. “Complex Disease of the Pleural Space: The 10 Questions Most Frequently Asked of the Radiologist–New Approaches to Their Answers With CT and MR Imaging.” Radiographics 17: 1043–1050. 10.1148/radiographics.17.4.9225404. 9225404

[cph470143-bib-0066] Lai‐Fook, S. J. , P. K. Houtz , and P. D. Jones . 2005. “Transdiaphragmatic Transport of Tracer Albumin From Peritoneal to Pleural Liquid Measured in Rats.” Journal of Applied Physiology 1985, no. 99: 2212–2221. 10.1152/japplphysiol.00731.2005. 16099890

[cph470143-bib-0067] Laine, G. A. , and S. J. Allen . 1991. “Left Ventricular Myocardial Edema. Lymph Flow, Interstitial Fibrosis, and Cardiac Function.” Circulation Research 68: 1713–1721. 10.1161/01.res.68.6.1713. 2036720

[cph470143-bib-0068] Leak, L. V. 1970. “Electron Microscopic Observations on Lymphatic Capillaries and the Structural Components of the Connective Tissue‐Lymph Interface.” Microvascular Research 2: 361–391. 10.1016/0026-2862(70)90031-2. 5523935

[cph470143-bib-0069] Leak, L. V. , and J. F. Burke . 1966. “Fine Structure of the Lymphatic Capillary and the Adjoining Connective Tissue Area.” American Journal of Anatomy 118: 785–809. 10.1002/aja.1001180308. 5956107

[cph470143-bib-0070] Leak, L. V. , and M. P. Jamuar . 1983. “Ultrastructure of Pulmonary Lymphatic Vessels.” American Review of Respiratory Disease 128: S59–S65. 10.1164/arrd.1983.128.2P2.S59. 6881711

[cph470143-bib-0071] Lee, G. M. , J. T. Stowell , K. Pope , B. W. Carter , and C. M. Walker . 2021. “Lymphatic Pathways of the Thorax: Predictable Patterns of Spread.” American Journal of Roentgenology 216: 649–658. 10.2214/AJR.20.23523. 33377793

[cph470143-bib-0072] Levick, J. , and N. McHale . 2003. “Lymph Productiona and Propulsion.” In Diseases of the Lymphatics, 44–64. Arnold.

[cph470143-bib-0073] Liedtke, W. , Y. Choe , M. A. Martí‐Renom , et al. 2000. “Vanilloid Receptor‐Related Osmotically Activated Channel (VR‐OAC), a Candidate Vertebrate Osmoreceptor.” Cell 103: 525–535. 10.1016/s0092-8674(00)00143-4. 11081638 PMC2211528

[cph470143-bib-0074] Liu, M.‐E. , B. F. Branstetter 4th , J. Whetstone , and E. J. Escott . 2006. “Normal CT Appearance of the Distal Thoracic Duct.” American Journal of Roentgenology 187: 1615–1620. 10.2214/AJR.05.1173. 17114559

[cph470143-bib-0075] Louveau, A. , I. Smirnov , T. J. Keyes , et al. 2015. “Structural and Functional Features of Central Nervous System Lymphatic Vessels.” Nature 523: 337–341. 10.1038/nature14432. 26030524 PMC4506234

[cph470143-bib-0076] Ludwig, L. L. , E. R. Schertel , J. W. Pratt , et al. 1997. “Impairment of Left Ventricular Function by Acute Cardiac Lymphatic Obstruction.” Cardiovascular Research 33: 164–171. 10.1016/s0008-6363(96)00177-0. 9059540

[cph470143-bib-0077] Maisel, K. , and H. Outtz Reed . 2025. “The Lymphatic Vasculature in Lung Homeostasis and Disease.” Annual Review of Physiology 87: 421–446. 10.1146/annurev-physiol-022724-105311. 39532108

[cph470143-bib-0078] Marcozzi, C. , A. Moriondo , E. Solari , et al. 2015. “Regional Lung Tissue Changes With Mechanical Ventilation and Fluid Load.” Experimental Lung Research 41: 228–240. 10.3109/01902148.2014.1003436. 25844691

[cph470143-bib-0079] Mathiisen, T. M. , K. P. Lehre , N. C. Danbolt , and O. P. Ottersen . 2010. “The Perivascular Astroglial Sheath Provides a Complete Covering of the Brain Microvessels: An Electron Microscopic 3D Reconstruction.” Glia 58: 1094–1103. 10.1002/glia.20990. 20468051

[cph470143-bib-0080] Mehlhorn, U. , H. J. Geissler , G. A. Laine , and S. J. Allen . 2001. “Myocardial Fluid Balance.” European Journal of Cardio‐Thoracic Surgery 20: 1220–1230. 10.1016/s1010-7940(01)01031-4. 11717032

[cph470143-bib-0081] Michel, C. C. , and M. E. Phillips . 1987. “Steady‐State Fluid Filtration at Different Capillary Pressures in Perfused Frog Mesenteric Capillaries.” Journal of Physiology 388: 421–435. 10.1113/jphysiol.1987.sp016622. 3498833 PMC1192556

[cph470143-bib-0082] Miserocchi, G. 2009. “Mechanisms Controlling the Volume of Pleural Fluid and Extravascular Lung Water.” European Respiratory Review 18: 244–252. 10.1183/09059180.00002709. 20956149

[cph470143-bib-0083] Miserocchi, G. , S. Kelly , and D. Negrini . 1988. “Pleural and Extrapleural Interstitial Liquid Pressure Measured by Cannulas and Micropipettes.” Journal of Applied Physiology 1985, no. 65: 555–562. 10.1152/jappl.1988.65.2.555. 3170405

[cph470143-bib-0084] Miserocchi, G. , E. Mariani , and D. Negrini . 1982. “Role of the Diaphragm in Setting Liquid Pressure in Serous Cavities.” Respiration Physiology 50: 381–392. 10.1016/0034-5687(82)90030-5. 7163656

[cph470143-bib-0085] Miserocchi, G. , T. Nakamura , E. Mariani , and D. Negrini . 1981. “Pleural Liquid Pressure Over the Interlobar Mediastinal and Diaphragmatic Surfaces of the Lung.” Respiration Physiology 46: 61–69. 10.1016/0034-5687(81)90068-2. 7330492

[cph470143-bib-0086] Miserocchi, G. , D. Negrini , and C. Gonano . 1990. “Direct Measurement of Interstitial Pulmonary Pressure in In Situ Lung With Intact Pleural Space.” Journal of Applied Physiology 1985, no. 69: 2168–2174. 10.1152/jappl.1990.69.6.2168. 2077013

[cph470143-bib-0087] Miserocchi, G. , D. Negrini , and C. Gonano . 1991. “Parenchymal Stress Affects Interstitial and Pleural Pressures in In Situ Lung.” Journal of Applied Physiology 1985, no. 71: 1967–1972. 10.1152/jappl.1991.71.5.1967. 1761498

[cph470143-bib-0088] Miserocchi, G. , G. Sancini , F. Mantegazza , and G. Chiappino . 2008. “Translocation Pathways for Inhaled Asbestos Fibers.” Environmental Health 7: 4. 10.1186/1476-069X-7-4. 18218073 PMC2265277

[cph470143-bib-0089] Miserocchi, G. , D. Venturoli , D. Negrini , and M. Del Fabbro . 1993. “Model of Pleural Fluid Turnover.” Journal of Applied Physiology (1985) 75: 1798–1806. 10.1152/jappl.1993.75.4.1798. 8282634

[cph470143-bib-0090] Moriondo, A. , F. Bianchin , C. Marcozzi , and D. Negrini . 2008. “Kinetics of Fluid Flux in the Rat Diaphragmatic Submesothelial Lymphatic Lacunae.” American Journal of Physiology. Heart and Circulatory Physiology 295: H1182–H1190. 10.1152/ajpheart.00369.2008. 18641277

[cph470143-bib-0091] Moriondo, A. , F. Boschetti , F. Bianchin , S. Lattanzio , C. Marcozzi , and D. Negrini . 2010. “Tissue Contribution to the Mechanical Features of Diaphragmatic Initial Lymphatics.” Journal of Physiology 588: 3957–3969. 10.1113/jphysiol.2010.196204. 20724369 PMC3000585

[cph470143-bib-0092] Moriondo, A. , A. Grimaldi , L. Sciacca , M. L. Guidali , C. Marcozzi , and D. Negrini . 2007. “Regional Recruitment of Rat Diaphragmatic Lymphatics in Response to Increased Pleural or Peritoneal Fluid Load.” Journal of Physiology 579: 835–847. 10.1113/jphysiol.2006.127126. 17218349 PMC2151369

[cph470143-bib-0093] Moriondo, A. , C. Marcozzi , F. Bianchin , et al. 2012. “Impact of Mechanical Ventilation and Fluid Load on Pulmonary Glycosaminoglycans.” Respiratory Physiology & Neurobiology 181: 308–320. 10.1016/j.resp.2012.03.013. 22484819

[cph470143-bib-0094] Moriondo, A. , S. Mukenge , and D. Negrini . 2005. “Transmural Pressure in Rat Initial Subpleural Lymphatics During Spontaneous or Mechanical Ventilation.” American Journal of Physiology. Heart and Circulatory Physiology 289: H263–H269. 10.1152/ajpheart.00060.2005. 15833809

[cph470143-bib-0095] Moriondo, A. , P. Pelosi , A. Passi , et al. 2007. “Proteoglycan Fragmentation and Respiratory Mechanics in Mechanically Ventilated Healthy Rats.” Journal of Applied Physiology 103: 747–756. 10.1152/japplphysiol.00056.2007. 17569774

[cph470143-bib-0096] Moriondo, A. , E. Solari , C. Marcozzi , and D. Negrini . 2013. “Spontaneous Activity in Peripheral Diaphragmatic Lymphatic Loops.” American Journal of Physiology. Heart and Circulatory Physiology 305: H987–H995. 10.1152/ajpheart.00418.2013. 23893166

[cph470143-bib-0097] Moriondo, A. , E. Solari , C. Marcozzi , and D. Negrini . 2015. “Diaphragmatic Lymphatic Vessel Behavior During Local Skeletal Muscle Contraction.” American Journal of Physiology. Heart and Circulatory Physiology 308: H193–H205. 10.1152/ajpheart.00701.2014. 25485903

[cph470143-bib-0098] Moriondo, A. , E. Solari , C. Marcozzi , and D. Negrini . 2016. “Lymph Flow Pattern in Pleural Diaphragmatic Lymphatics During Intrinsic and Extrinsic Isotonic Contraction.” American Journal of Physiology. Heart and Circulatory Physiology 310: H60–H70. 10.1152/ajpheart.00640.2015. 26519032

[cph470143-bib-0099] Mukenge, S. , D. Negrini , and O. Alfieri . 2023. “Secondary Lymphedema: Clinical Interdisciplinary Tricks to Overcome an Intriguing Disease.” Biology 12: 646. 10.3390/biology12050646. 37237460 PMC10215137

[cph470143-bib-0100] Mukenge, S. , D. Negrini , M. Catena , et al. 2013. “Development of Functionally Patent Lymphatic Meshes in Postsurgical Long‐Term Resolution of Peripheral Secondary Lymphedema.” Journal of Vascular Surgery. Venous and Lymphatic Disorders 1: 280–288. 10.1016/j.jvsv.2012.09.008. 26992588

[cph470143-bib-0101] Mukenge, S. , C. Pulitanò , R. Colombo , D. Negrini , and G. Ferla . 2007. “Secondary Scrotal Lymphedema: A Novel Microsurgical Approach.” Microsurgery 27: 655–656. 10.1002/micr.20426. 17929261

[cph470143-bib-0102] Nahrendorf, M. , F. K. Swirski , E. Aikawa , et al. 2007. “The Healing Myocardium Sequentially Mobilizes Two Monocyte Subsets With Divergent and Complementary Functions.” Journal of Experimental Medicine 204: 3037–3047. 10.1084/jem.20070885. 18025128 PMC2118517

[cph470143-bib-0103] Negrini, D. 2006. “Lymph Flow Modulation: The Tricks of a Performant Machinery.” Journal of Physiology 575: 687. 10.1113/jphysiol.2006.116293. 16825294 PMC1995681

[cph470143-bib-0104] Negrini, D. 2022. “Morphological, Mechanical and Hydrodynamic Aspects of Diaphragmatic Lymphatics.” Biology 11: 1803. 10.3390/biology11121803. 36552311 PMC9775868

[cph470143-bib-0105] Negrini, D. , S. T. Ballard , and J. N. Benoit . 1994. “Contribution of Lymphatic Myogenic Activity and Respiratory Movements to Pleural Lymph Flow.” Journal of Applied Physiology 1985, no. 76: 2267–2274. 10.1152/jappl.1994.76.6.2267. 7928846

[cph470143-bib-0106] Negrini, D. , and M. Del Fabbro . 1999. “Subatmospheric Pressure in the Rabbit Pleural Lymphatic Network.” Journal of Physiology 520, no. Pt 3: 761–769. 10.1111/j.1469-7793.1999.00761.x. 10545142 PMC2269608

[cph470143-bib-0107] Negrini, D. , M. Del Fabbro , C. Gonano , S. Mukenge , and G. Miserocchi . 1992. “Distribution of Diaphragmatic Lymphatic Lacunae.” Journal of Applied Physiology 1985, no. 72: 1166–1172. 10.1152/jappl.1992.72.3.1166. 1568971

[cph470143-bib-0108] Negrini, D. , M. del Fabbro , and D. Venturoli . 1993. “Fluid Exchanges Across the Parietal Peritoneal and Pleural Mesothelia.” Journal of Applied Physiology 1985, no. 74: 1779–1784. 10.1152/jappl.1993.74.4.1779. 8514696

[cph470143-bib-0109] Negrini, D. , C. Gonano , M. Del Fabbro , and G. Miserocchi . 1990. “Transperitoneal Fluid Dynamics in Rabbit Liver.” Journal of Applied Physiology 1985, no. 69: 625–629. 10.1152/jappl.1990.69.2.625. 2228874

[cph470143-bib-0110] Negrini, D. , C. Gonano , and G. Miserocchi . 1992. “Microvascular Pressure Profile in intact In Situ Lung.” Journal of Applied Physiology 1985, no. 72: 332–339. 10.1152/jappl.1992.72.1.332. 1537734

[cph470143-bib-0111] Negrini, D. , C. Marcozzi , E. Solari , et al. 2016. “Hyperpolarization‐Activated Cyclic Nucleotide‐Gated Channels in Peripheral Diaphragmatic Lymphatics.” American Journal of Physiology. Heart and Circulatory Physiology 311: H892–H903. 10.1152/ajpheart.00193.2016. 27496876

[cph470143-bib-0112] Negrini, D. , and G. Miserocchi . 1989. “Size‐Related Differences in Parietal Extrapleural and Pleural Liquid Pressure Distribution.” Journal of Applied Physiology 1985, no. 67: 1967–1972. 10.1152/jappl.1989.67.5.1967. 2600028

[cph470143-bib-0113] Negrini, D. , A. Moriondo , and S. Mukenge . 2004. “Transmural Pressure During Cardiogenic Oscillations in Rodent Diaphragmatic Lymphatic Vessels.” Lymphatic Research and Biology 2: 69–81. 10.1089/lrb.2004.2.69. 15615488

[cph470143-bib-0114] Negrini, D. , S. Mukenge , M. Del Fabbro , C. Gonano , and G. Miserocchi . 1991. “Distribution of Diaphragmatic Lymphatic Stomata.” Journal of Applied Physiology 1985, no. 70: 1544–1549. 10.1152/jappl.1991.70.4.1544. 2055834

[cph470143-bib-0115] Negrini, D. , A. Passi , and A. Moriondo . 2008. “The Role of Proteoglycans in Pulmonary Edema Development.” Intensive Care Medicine 34: 610–618. 10.1007/s00134-007-0962-y. 18264693

[cph470143-bib-0116] Negrini, D. , M. Pistolesi , M. Miniati , R. Bellina , C. Giuntini , and G. Miserocchi . 1985. “Regional Protein Absorption Rates From the Pleural Cavity in Dogs.” Journal of Applied Physiology 1985, no. 58: 2062–2067. 10.1152/jappl.1985.58.6.2062. 4008421

[cph470143-bib-0117] Negrini, D. , O. Tenstad , A. Passi , and H. Wiig . 2006. “Differential Degradation of Matrix Proteoglycans and Edema Development in Rabbit Lung.” American Journal of Physiology. Lung Cellular and Molecular Physiology 290: L470–L477. 10.1152/ajplung.00310.2005. 16214813

[cph470143-bib-0118] Nilsson, J. C. , G. Nielsen , B. A. Groenning , et al. 2001. “Sustained Postinfarction Myocardial Oedema in Humans Visualised by Magnetic Resonance Imaging.” Heart 85: 639–642. 10.1136/heart.85.6.639. 11359743 PMC1729755

[cph470143-bib-0119] Nishii, T. , A. K. Kono , M. Shigeru , et al. 2014. “Cardiovascular Magnetic Resonance T2 Mapping Can Detect Myocardial Edema in Idiopathic Dilated Cardiomyopathy.” International Journal of Cardiovascular Imaging 30: 65–72. 10.1007/s10554-014-0414-z. 24715436

[cph470143-bib-0120] Nomoto, Y. , T. Suga , K. Nakajima , et al. 1989. “Acute Hydrothorax in Continuous Ambulatory Peritoneal Dialysis–A Collaborative Study of 161 Centers.” American Journal of Nephrology 9: 363–367. 10.1159/000167997. 2679094

[cph470143-bib-0121] Okiemy, G. , C. Foucault , C. Avisse , G. Hidden , and M. Riquet . 2003. “Lymphatic Drainage of the Diaphragmatic Pleura to the Peritracheobronchial Lymph Nodes.” Surgical and Radiologic Anatomy 25: 32–35. 10.1007/s00276-002-0081-y. 12677463

[cph470143-bib-0122] Patlak, C. S. , D. A. Goldstein , and J. F. Hoffman . 1963. “The Flow of Solute and Solvent Across a Two‐Membrane System.” Journal of Theoretical Biology 5: 426–442. 10.1016/0022-5193(63)90088-2. 5875168

[cph470143-bib-0123] Pelosi, P. , and D. Negrini . 2008. “Extracellular Matrix and Mechanical Ventilation in Healthy Lungs: Back to Baro/Volotrauma?” Current Opinion in Critical Care 14: 16–21. 10.1097/MCC.0b013e3282f25162. 18195621

[cph470143-bib-0124] Pollay, M. 2010. “The Function and Structure of the Cerebrospinal Fluid Outflow System.” Cerebrospinal Fluid Research 7: 9. 10.1186/1743-8454-7-9. 20565964 PMC2904716

[cph470143-bib-0125] Pujari, A. , A. F. Smith , J. D. Hall , et al. 2020. “Lymphatic Valves Separate Lymph Flow Into a Central Stream and a Slow‐Moving Peri‐Valvular Milieu.” Journal of Biomechanical Engineering 142: 100805. 10.1115/1.4048028. 32766737 PMC7477708

[cph470143-bib-0126] Raj, J. U. , and P. Chen . 1986. “Microvascular Pressures Measured by Micropuncture in Isolated Perfused Lamb Lungs.” Journal of Applied Physiology 1985, no. 61: 2194–2201. 10.1152/jappl.1986.61.6.2194. 3804926

[cph470143-bib-0127] Reed, H. O. , L. Wang , J. Sonett , et al. 2019. “Lymphatic Impairment Leads to Pulmonary Tertiary Lymphoid Organ Formation and Alveolar Damage.” Journal of Clinical Investigation 129: 2514–2526. 10.1172/JCI125044. 30946031 PMC6546450

[cph470143-bib-0128] Rippe, B. , and B. Haraldsson . 1994. “Transport of Macromolecules Across Microvascular Walls: The Two‐Pore Theory.” Physiological Reviews 74: 163–219. 10.1152/physrev.1994.74.1.163. 8295933

[cph470143-bib-0129] Riquet, M. , F. Le Pimpec Barthes , R. Souilamas , and G. Hidden . 2002. “Thoracic Duct Tributaries From Intrathoracic Organs.” Annals of Thoracic Surgery 73: 892–898. 10.1016/s0003-4975(01)03361-6. 11899197

[cph470143-bib-0130] Rousselle, P. , D. R. Keene , F. Ruggiero , M. F. Champliaud , M. Rest , and R. E. Burgeson . 1997. “Laminin 5 Binds the NC‐1 Domain of Type VII Collagen.” Journal of Cell Biology 138: 719–728. 10.1083/jcb.138.3.719. 9245798 PMC2141627

[cph470143-bib-0131] Schmid‐Schönbein, G. W. 1990. “Microlymphatics and Lymph Flow.” Physiological Reviews 70: 987–1028. 10.1152/physrev.1990.70.4.987. 2217560

[cph470143-bib-0132] Schraufnagel, D. E. 2013. “Lung Lymphatics: Why Should a Clinician Care?” Annals of the American Thoracic Society 10: 148–149. 10.1513/AnnalsATS.201302-029ED. 23607845

[cph470143-bib-0133] Skalak, T. C. , G. W. Schmid‐Schönbein , and B. W. Zweifach . 1984. “New Morphological Evidence for a Mechanism of Lymph Formation in Skeletal Muscle.” Microvascular Research 28: 95–112. 10.1016/0026-2862(84)90032-3. 6748962

[cph470143-bib-0134] Solari, E. , C. Marcozzi , B. Bartolini , M. Viola , D. Negrini , and A. Moriondo . 2020. “Acute Exposure of Collecting Lymphatic Vessels to Low‐Density Lipoproteins Increases Both Contraction Frequency and Lymph Flow: An In Vivo Mechanical Insight.” Lymphatic Research and Biology 18: 146–155. 10.1089/lrb.2019.0040. 31526222

[cph470143-bib-0135] Solari, E. , C. Marcozzi , M. Bistoletti , et al. 2020. “TRPV4 Channels' Dominant Role in the Temperature Modulation of Intrinsic Contractility and Lymph Flow of Rat Diaphragmatic Lymphatics.” American Journal of Physiology. Heart and Circulatory Physiology 319: H507–H518. 10.1152/ajpheart.00175.2020. 32706268

[cph470143-bib-0136] Solari, E. , C. Marcozzi , D. Negrini , and A. Moriondo . 2017. “Temperature‐Dependent Modulation of Regional Lymphatic Contraction Frequency and Flow.” American Journal of Physiology. Heart and Circulatory Physiology 313: H879–H889. 10.1152/ajpheart.00267.2017. 28778912

[cph470143-bib-0137] Solari, E. , C. Marcozzi , D. Negrini , and A. Moriondo . 2018. “Fluid Osmolarity Acutely and Differentially Modulates Lymphatic Vessels Intrinsic Contractions and Lymph Flow.” Frontiers in Physiology 9: 871. 10.3389/fphys.2018.00871. 30026707 PMC6041695

[cph470143-bib-0138] Solari, E. , C. Marcozzi , D. Negrini , and A. Moriondo . 2020. “Lymphatic Vessels and Their Surroundings: How Local Physical Factors Affect Lymph Flow.” Biology 9: 463. 10.3390/biology9120463. 33322476 PMC7763507

[cph470143-bib-0139] Solari, E. , C. Marcozzi , D. Negrini , and A. Moriondo . 2023. “Fluid Osmolarity Modulates the Rate of Spontaneous Contraction of Lymphatic Vessels and Lymph Flow by Means of a Cooperation Between TRPV and VRAC Channels.” Biology 12: 1039. 10.3390/biology12071039. 37508468 PMC10376700

[cph470143-bib-0140] Solari, E. , C. Marcozzi , D. Negrini , and A. Moriondo . 2026. “The “Useful” Hindrance to Flow: Quantification of Intraluminal Valves Effect on Lymph Flow Driven by Intrinsic Mechanism in the Diaphragmatic Lymphatic Network.” American Journal of Physiology. Heart and Circulatory Physiology 330: H388–H403. 10.1152/ajpheart.00759.2025. 41442184

[cph470143-bib-0141] Solari, E. , C. Marcozzi , C. Ottaviani , D. Negrini , and A. Moriondo . 2022. “Draining the Pleural Space: Lymphatic Vessels Facing the Most Challenging Task.” Biology 11: 419. 10.3390/biology11030419. 35336793 PMC8945018

[cph470143-bib-0142] Song, L. , X. Chen , T. A. Swanson , et al. 2020. “Lymphangiogenic Therapy Prevents Cardiac Dysfunction by Ameliorating Inflammation and Hypertension.” eLife 9: e58376. 10.7554/eLife.58376. 33200983 PMC7695461

[cph470143-bib-0143] Starling, E. 1896. “Absorption of Fluid From Connective Tissue Spaces.” Journal of Physiology 19: 312–326. 10.1113/jphysiol.1896.sp000596 PMC1512609 16992325

[cph470143-bib-0144] Strotmann, R. , C. Harteneck , K. Nunnenmacher , G. Schultz , and T. D. Plant . 2000. “OTRPC4, a Nonselective Cation Channel That Confers Sensitivity to Extracellular Osmolarity.” Nature Cell Biology 2: 695–702. 10.1038/35036318. 11025659

[cph470143-bib-0145] Tatin, F. , E. Renaud‐Gabardos , A.‐C. Godet , et al. 2017. “Apelin Modulates Pathological Remodeling of Lymphatic Endothelium After Myocardial Infarction.” JCI Insight 2: 93887. 10.1172/jci.insight.93887. 28614788 PMC5470877

[cph470143-bib-0146] Taylor, A. , and J. Parker . 1985. “Pulmonary Interstitial Space and Lymphatics.” In Handbook of Physiology. The Respiratory System. Circulations and Non Respiratory Functions, 167–230. American Physiological Society.

[cph470143-bib-0147] Telinius, N. , J. Majgaard , S. Kim , et al. 2015. “Voltage‐Gated Sodium Channels Contribute to Action Potentials and Spontaneous Contractility in Isolated Human Lymphatic Vessels.” Journal of Physiology 593: 3109–3122. 10.1113/JP270166. 25969124 PMC4532530

[cph470143-bib-0148] Telinius, N. , S. Mohanakumar , J. Majgaard , et al. 2014. “Human Lymphatic Vessel Contractile Activity Is Inhibited In Vitro but Not In Vivo by the Calcium Channel Blocker Nifedipine.” Journal of Physiology 592: 4697–4714. 10.1113/jphysiol.2014.276683. 25172950 PMC4253471

[cph470143-bib-0149] Trapnell, D. H. 1970. “The Anatomy of the Lymphatics of the Lungs and Chest Wall.” Thorax 25: 255–256. 10.1136/thx.25.2.255-d. PMC472162 5427362

[cph470143-bib-0150] Trzewik, J. , S. K. Mallipattu , G. M. Artmann , F. A. Delano , and G. W. Schmid‐Schönbein . 2001. “Evidence for a Second Valve System in Lymphatics: Endothelial Microvalves.” FASEB 15: 1711–1717. 10.1096/fj.01-0067com. 11481218

[cph470143-bib-0151] van Amerongen, M. J. , M. C. Harmsen , N. van Rooijen , A. H. Petersen , and M. J. A. van Luyn . 2007. “Macrophage Depletion Impairs Wound Healing and Increases Left Ventricular Remodeling After Myocardial Injury in Mice.” American Journal of Pathology 170: 818–829. 10.2353/ajpath.2007.060547. 17322368 PMC1864893

[cph470143-bib-0152] Van Helden, D. F. 1993. “Pacemaker Potentials in Lymphatic Smooth Muscle of the Guinea‐Pig Mesentery.” Journal of Physiology 471: 465–479. 10.1113/jphysiol.1993.sp019910. 8120817 PMC1143971

[cph470143-bib-0153] Verbrugge, F. H. , P. B. Bertrand , E. Willems , et al. 2017. “Global Myocardial Oedema in Advanced Decompensated Heart Failure.” European Heart Journal Cardiovascular Imaging 18: 787–794. 10.1093/ehjci/jew131. 27378769

[cph470143-bib-0154] Vieira, J. M. , S. Norman , C. Villa Del Campo , et al. 2018. “The Cardiac Lymphatic System Stimulates Resolution of Inflammation Following Myocardial Infarction.” Journal of Clinical Investigation 128: 3402–3412. 10.1172/JCI97192. 29985167 PMC6063482

[cph470143-bib-0155] von der Weid, P. Y. 1998. “ATP‐Sensitive K+ Channels in Smooth Muscle Cells of Guinea‐Pig Mesenteric Lymphatics: Role in Nitric Oxide and Beta‐Adrenoceptor Agonist‐Induced Hyperpolarizations.” British Journal of Pharmacology 125: 17–22. 10.1038/sj.bjp.0702026. 9776338 PMC1565588

[cph470143-bib-0156] von der Weid, P.‐Y. , and D. C. Zawieja . 2004. “Lymphatic Smooth Muscle: The Motor Unit of Lymph Drainage.” International Journal of Biochemistry & Cell Biology 36: 1147–1153. 10.1016/j.biocel.2003.12.008. 15109561

[cph470143-bib-0157] Wang, N. S. 1975. “The Preformed Stomas Connecting the Pleural Cavity and the Lymphatics in the Parietal Pleura.” American Review of Respiratory Disease 111: 12–20. 10.1164/arrd.1975.111.1.12. 1111395

[cph470143-bib-0158] Wang, N. S. 1985. “Anatomy and Physiology of the Pleural Space.” Clinics in Chest Medicine 6: 3–16. 3891209

[cph470143-bib-0159] Wang, Q. X. , O. Ohtani , M. Saitoh , and Y. Ohtani . 1997. “Distribution and Ultrastructure of the Stomata Connecting the Pleural Cavity With Lymphatics in the Rat Costal Pleura.” Acta Anatomica (Basel) 158: 255–265. 10.1159/000147938. 9416356

[cph470143-bib-0160] Ware, S. A. , L. Zheng , M. Almasian , et al. 2026. “Normal Cardiac Lymphatics and Their Mimics.” American Journal of Physiology. Heart and Circulatory Physiology 330, no. 1: H170–H186. 41285409 10.1152/ajpheart.00549.2025 PMC12758634

[cph470143-bib-0161] Wu, L. , X. Gao , R. C. Brown , S. Heller , and R. G. O'Neil . 2007. “Dual Role of the TRPV4 Channel as a Sensor of Flow and Osmolality in Renal Epithelial Cells.” American Journal of Physiology. Renal Physiology 293: F1699–F1713. 10.1152/ajprenal.00462.2006. 17699550

[cph470143-bib-0162] Zakharov, A. , C. Papaiconomou , J. Djenic , R. Midha , and M. Johnston . 2003. “Lymphatic Cerebrospinal Fluid Absorption Pathways in Neonatal Sheep Revealed by Subarachnoid Injection of Microfil.” Neuropathology and Applied Neurobiology 29: 563–573. 10.1046/j.0305-1846.2003.00508.x. 14636163

[cph470143-bib-0163] Zawieja, D. C. 2009. “Contractile Physiology of Lymphatics.” Lymphatic Research and Biology 7: 87–96. 10.1089/lrb.2009.0007. 19534632 PMC2925033

[cph470143-bib-0164] Zhang, Q. , Y. Niu , Y. Li , et al. 2025. “Meningeal Lymphatic Drainage: Novel Insights Into Central Nervous System Disease.” Signal Transduction and Targeted Therapy 10: 142. 10.1038/s41392-025-02177-z. 40320416 PMC12050339

